# Photocatalytic proximity labelling of MCL-1 by a BH3 ligand

**DOI:** 10.1038/s42004-019-0235-z

**Published:** 2019-11-21

**Authors:** Hester A. Beard, Jacob R. Hauser, Martin Walko, Rachel M. George, Andrew J. Wilson, Robin S. Bon

**Affiliations:** 1 School of Chemistry, University of Leeds, Woodhouse Lane, Leeds LS2 9JT, UK; 2 Astbury Centre for Structural Molecular Biology, University of Leeds, Woodhouse Lane, Leeds LS2 9JT, UK; 3 Leeds Institute of Cardiovascular and Metabolic Medicine, LIGHT laboratories, University of Leeds, Leeds LS2 9JT, UK

## Abstract

Ligand-directed protein labelling allows the introduction of diverse chemical functionalities onto proteins without the need for genetically encoded tags. Here we report a method for the rapid labelling of a protein using a ruthenium-bipyridyl (Ru(II)(bpy)_3_)-modified peptide designed to mimic an interacting BH3 ligand within a BCL-2 family protein-protein interactions. Using sub-stoichiometric quantities of (Ru(II)(bpy)_3_)-modified NOXA-B and irradiation with visible light for 1 min, the anti-apoptotic protein MCL-1 can be photolabelled with a variety of functional tags. In contrast with previous reports on Ru(II)(bpy)_3_-mediated photolabelling, tandem mass spectrometry experiments reveal that the labelling site is a cysteine residue of MCL-1. MCL-1 can be labelled selectively in mixtures with other proteins, including the structurally related BCL-2 member, BCL-x_L_. These results demonstrate that proximity-induced photolabelling is applicable to interfaces that mediate protein-protein interactions, and pave the way towards future use of ligand-directed proximity labelling for dynamic analysis of the interactome of BCL-2 family proteins.

Most cellular proteins function as dynamic complexes with other proteins and, conversely, protein-protein interactions (PPIs) play key roles in the regulation of most biological processes^1^. While stable PPIs are usually associated with multi-subunit protein complexes and quaternary structure, transient PPIs regulate multiple cellular processes, and are implicated in a variety of disease states^2^. Ongoing efforts to study transient PPIs may lead to better understanding of the processes of life and the development of novel diagnostics and therapeutics. As part of such efforts, the ability to selectively introduce chemical labels onto proteins involved in specific PPIs would provide new tools to study such PPIs, including the construction of protein-based biosensors or affinity enrichment reagents for dynamic interactome analysis.

Although numerous methods for chemical protein labelling exist, only few are suitable for the selective labelling of (subsets of) native proteins in complex biological samples, such as cell lysates, whole cells and tissues^3^. Many of these methods rely on enzymatic activity for the labelling of highly nucleophilic active site residues^4^ or on the metabolic incorporation of non-natural substrates, such as amino acids^5,6^, carbohydrates^7^ and lipids^8,9^, which can be further derivatised through bio-orthogonal chemistry^5,10,11^. An effective strategy that does not rely on enzymatic activity or post-translational modifications, and would therefore be especially suitable for the study of PPIs, is the use of ligand-directed protein labelling (LDL)^12,13^. LDL relies on reagents consisting of a ligand for the protein-of-interest attached to a (moderately) reactive chemical group (exchange/cleavage approach) or to a catalyst that activates a third component (catalyst tethering approach). Upon binding to its target protein, these LDL reagents cause the transfer of chemical labels to specific, proximal amino acid residues. Hamachi and co-workers have developed a wide range of LDL methods capable of selectively modifying native proteins in a ‘traceless’ manner, in which the ligand leaves its binding site after the labelling reaction and the protein is able to perform its native function^14^. These methods include exchange/cleavage approaches based on electrophilic phenylsulfonate esters^15–17^, acyl imidazoles^18,19^, *N*-sul-fonyl pyridines^20^, or *N*-acyl-*N*-alkyl sulfonamides^21^ and catalyst tethering approaches based on *N*, *N*-dimethylaminopyridine (DMAP)^22–24^ or oxime reagents^25^. In addition, several groups have developed LDL reagents incorporating transition metal catalysts. Ball and co-workers used rhodium(II) metallopeptides to selectively modify side-chains on protein surfaces with functionalised diazo compounds^26–29^. Based on chemistry established by Kodadek et al.^30–32^, the group of Nakamura developed a photocatalytic LDL method based on local single-electron transfer (SET) mediated by a Ru(II)(bpy)_3_ photocatalyst tethered to a small molecule (Fig. 1a)^33–37^. Irradiation of the ruthenium complex with visible light results in an excited state [Ru(II) (bpy)_3_]* complex of relatively long lifetime (~1 ms) that can function as electron donor or electron acceptor^38^. In photo-catalytic LDL, the excited [Ru(II)(bpy)_3_]* moiety loses an electron to a sacrificial oxidant such as ammonium persulfate (APS) or molecular oxygen^31^, and then catalyses reactions between tyrosine residues proximal to the ligand-binding site and electronrich dimethylaniline^33–35^ or 1-methyl-4-aryl-urazole (MAUra)^36^ derivatives that may act as radical trapping agents (RTAs; e.g., carrying fluorescent or biotinylated labels; Fig. 1a). Photocatalytic LDL reagents based on the ligands benzenesulfonamide, gefitinib and methotrexate were used to label or immobilise carbonic anhydrase II (CAII)^33,35,36^, epidermal growth factor receptor (EGFR)^35^ or dihydrofolate reductase (DHFR)^34^, respectively. However, it should be noted that excesses of the ligand-directed Ru(II)(bpy)_3_ catalyst were employed in the aforementioned studies, meaning the catalytic potential of this labelling chemistry has not yet been demonstrated.

The majority of LDL approaches are based on interactions of proteins with small-molecule ligands. In contrast, the scope of LDL within the context of PPIs is less well developed, and only few examples use peptides as the ligand component of LDL reagents^24,26–28,39^. Given a significant proportion of PPIs are mediated by peptide interacting motifs^40^, we sought to exploit peptides for the development of LDL reagents for selective labelling of proteins involved in transient PPIs. To enable future studies of PPIs that may require temporal control in the identification, visualisation or perturbation of transient PPIs, we chose to base our LDL approach on [Ru(II)(bpy)_3_]-mediated photocatalysis. As a model system, we chose proteins of the B-cell lymphoma 2 (BCL-2) family. BCL-2 proteins regulate apoptotic cell death in response to pro- and anti-apoptotic signals through a variety of transient PPIs between pro-apoptotic (e.g., BAK, BAX), anti-apoptotic (e.g., BCL-2, BCL-x_L_, MCL-1) and effector (e.g., BID, BIM, PUMA, NOXA-B) members of the BCL-2 family^41^. PPIs in this family are mediated by the binding of a BH3 domain—in a helical conformation—of effector/pro-apoptotic BCL-2 family members to a groove on the surface of anti-apoptotic partners. The exact mechanisms via which apoptosis is regulated by PPIs of the BCL-2 family are not yet fully understood, but anti-apoptotic members are over-expressed in certain cancers. Notably, MCL-1 has come into focus as a small-molecule anticancer target in its own right, due to its role in resistance to approved anticancer therapies^42–44^. To enable studies of BCL-2 family PPIs, in this work our objective was to develop the underlying methodology for selective chemical labelling of native (unmodified) BCL-2 family proteins.

Here, we report the rapid and selective photolabelling of MCL-1 using a NOXA-B BH3 peptide incorporating an *N*-terminal Ru (II)(bpy)_3_ substituent and designed to mimic an interacting BH3 ligand within a BCL-2 family protein-protein interactions (Fig. 1b). We show that sub-stoichiometric quantities of (Ru(II) (bpy)_3_)-modified NOXA-B and irradiation with visible light for 1 min can be used to selectively label MCL-1 in mixtures of proteins, and that, in contrast to previous reports on SET-mediated LDL, labelling occurs on a specific cysteine residue.

## Results

### Design and synthesis of reagents

**Fig. 2:**
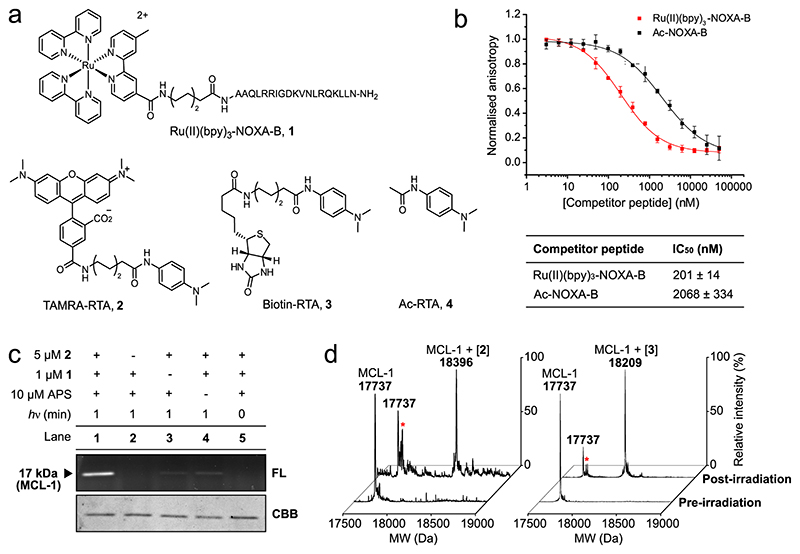
Ligand-directed labelling of MCL-1 with fluorescent and biotinylated radical trapping agents. RTA: radical trapping agent. **a** Chemical structures of ligand-directed labelling reagent Ru(II)(bpy)_3_-NOXA-B **1**, fluorescent label TAMRA-RTA **2**, biotinylated label biotin-RTA **3** and minimal label Ac-RTA **4**. **b** Fluorescence anisotropy competition experiments for the inhibition of the FITC-NOXA-B/MCL-1 interaction by Ru(II)(bpy)_3_-NOXA-B **1** (red) and Ac-NOXA-B **5** (black). Error bars represent the standard deviation of three repeats. IC_50_ values for peptides **1** and **5** obtained from these experiments are quoted ± SEM. **c** Fluorescence image (FL) and Coomassie Brilliant Blue (CBB) stained SDS-PAGE gels of photolabelled mixtures; fluorescence image shows labelling of MCL-1 using 1 min irradiation, 5 μM TAMRA-RTA **2**, 1 μM Ru(II)(bpy)_3_-NOXA-B **1** and 10 μM APS; CBB stain shows MCL-1 for all conditions. Reactions were carried out on 5 μM MCL-1 in ammonium bicarbonate buffer, pH 7.4. Pictures of complete gels are shown in Supplementary Fig. 15. **d** Deconvoluted ESI-MS spectra of photolabelling reaction mixtures displaying fluorescently modified (18396 Da) and biotinylated MCL-1 (18209 Da). Star denotes possible oxidised protein species (see Supplementary Fig. 16 for annotated spectrum).

The BH3 domain NOXA-B_75-93(C75A)_ modified with an *N*-terminal FITC group (FITC-Ahx-AAQLRRIGDKVNLRQKLLN-CONH_2_; FITC-NOXA-B) retains its affinity for MCL-1, as determined by fluorescence anisotropy experiments^45^. Therefore, we designed an LDL reagent for the labelling of MCL-1 consisting of the same BH3 sequence linked to a Ru(II)(bpy)_3_ photocatalyst via an aminohexanoic acid (Ahx) linker on its *N*-terminus (Ru(II)(bpy)_3_-NOXA-B **1**; Fig. 2a). LDL reagent **1** and Ac-NOXA-B **5** were prepared through solid-phase peptide synthesis (Supplementary Figs. 1 and 2) and purified by preparative HPLC (Supplementary Figs. 3-6; Supplementary Tables 1 and 2). Recombinant MCL-1_172–327_ was expressed and purified as published previously^45^. Fluorescence anisotropy competition experiments—with FITC-NOXA-B as tracer—confirmed that **1** is a more potent inhibitor of the FITC-NOXA-B/MCL-1 interaction than the wild-type peptide Ac-NOXA-B **5** (as seen by a 10-fold reduction in IC_50_ value; Fig. 2b; Supplementary Fig. 11), which could be the result of the hydrophobic nature of the bpy ligands or the additional charge of the Ru(II)(bpy)_3_ substituent of **1**. Fluorescent (TAMRA-RTA **2**), biotinylated (biotin-RTA **3**) and minimal (Ac-RTA **4**) RTAs analogous to those developed by Nakamura and co-workers were synthesised through adaptation of literature procedures (Fig. 2a, Supplementary Figs. 9 and 10).

### Photocatalytic labelling of MCL-1

For photocatalytic LDL, Ru (II)(bpy)_3_-NOXA-B **1**, TAMRA-RTA **2** and ammonium persulfate (APS) were added to a buffered solution of MCL-1 and the mixture was irradiated for 1 min at 450 nm using blue LED lamps (see Supplementary Methods for details and Supplementary Fig. 14 for an overview of the setup). An aqueous solution of the radical scavenger dithiothreitol (DTT, 10 mM) was then added to quench the reaction^33^. Optimisation of the labelling conditions resulted in fluorescent modification of MCL-1 using 20 mol% of peptide-catalyst **1** and an equimolar concentration (relative to [MCL-1]) of TAMRA-RTA **2** (lane 1, Fig. 2c; Supplementary Fig. 15). Analysis of the labelled mixture using in-gel fluorescence indicated that ruthenium-modified peptide **1**, fluorescent RTA **2** and visible light irradiation were all necessary for efficient labelling of MCL-1. A small amount of background labelling of MCL-1 occurred in the absence of **1**, possibly due to the ability of the rhodamine dye in **2** to act as a photoredox catalyst^46,47^ (lane 3, Fig. 2c; more clearly seen when larger amounts of protein were loaded onto the SDS-PAGE gel: Supplementary Figs. 32 and 33). This explanation is consistent with the absence of background labelling when biotin-RTA **3** was used instead of TAMRA-RTA **2** (see below and Supplementary Fig. 18). Furthermore, the addition of APS to the reaction mixture was necessary for efficient labelling of MCL-1 in these experiments, despite a small amount of labelling occurring when APS was omitted (lane 4, Fig. 2c).

The identity of the labelled species was confirmed by intact protein electrospray ionisation mass spectrometry (ESI-MS). Prior to irradiation, only unmodified MCL-1 (17737 Da, Fig. 2d, left) could be detected in the reaction mixture. However, upon irradiation with visible light for 1 min, the mass spectrum of the reaction mixture showed peaks corresponding to both unmodified MCL-1 (17737 Da) and the labelled species [(MCL-1) + **2**] (18396 Da) (Fig. 2d, left). These data confirm the main reaction product results from the addition of one TAMRA-RTA label, suggesting labelling of a single amino acid residue on MCL-1. Several additional peaks were evident in the MS trace of the irradiated sample (denoted by a star, Fig. 2d), most likely resulting from the oxidation of amino acid residues on MCL-1 proximal to the Ru(II)(bpy)_3_ complex (indicated by several mass increases of +16 Da, see Supplementary Fig. 16). Oxidation of proteins using Ru(II)(bpy)_3_ reagents in the absence of a ‘radical trapper’ has been reported previously^31^, and this has been exploited in chromophore-assisted light inactivation (CALI)^48,49^ reagents that allow targeted protein inactivation with visible light, in vitro and in cells^35,37,50,51^. Similar results were obtained in LDL experiments with Ac-RTA **4**. The use of 20 mol% Ru(II) (bpy)_3_-NOXA-B **1** gave the highest yield of labelled MCL-1 (incorporating a single modification with Ac-RTA **4**), while the use of >20 mol% of **1** resulted in increased degradation of both MCL-1 and labelled MCL-1, even in the presence of an excess (10 molar equivalents with respect to MCL-1) of Ac-RTA **4** (Supplementary Fig. 17).

Next, we investigated the labelling of MCL-1 using a different functional tag, biotin-RTA **3**. ESI-MS analysis of the crude reaction mixture revealed the appearance of a distinct species (18209 Da, Fig. 2d, right) suggesting single biotin labelling of MCL-1 (for control experiments in the absence of individual reagents/conditions, see Supplementary Fig. 18). The biotinylated MCL-1 could be affinity-purified with avidin-agarose beads, demonstrating that the biotin itself had not been oxidised and was accessible (Supplementary Fig. 19). Although the ESI-mass spectrum appears cleaner than that of TAMRA-labelled MCL-1, irradiation times greater than 1 min resulted in higher conversion to oxidised protein species—of both unmodified and modified MCL-1 (Supplementary Fig. 20). Therefore, irradiation times should be kept to a maximum of 1 min to prevent oxidative damage of the labelled protein, and may need optimisation for specific protein-reagent pairs, sample type and light source. Taken together, these results suggest that substoichiometric Ru (II)(bpy)_3_-NOXA-B **1** can be used for the rapid and efficient photolabelling of MCL-1 with various reporter groups.

### Determination of the site of labelling on MCL-1

**Fig. 3:**
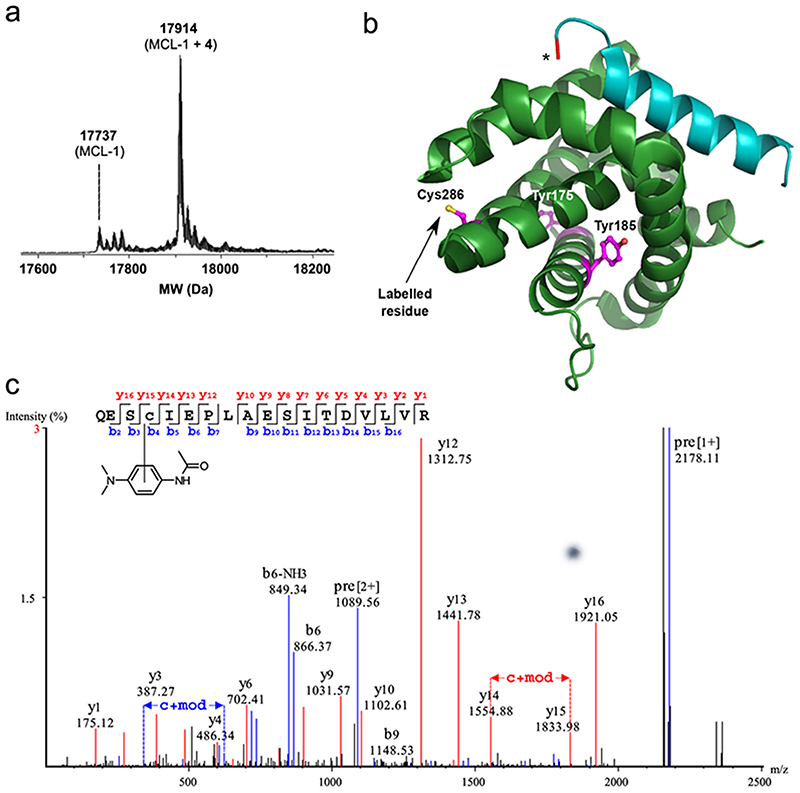
Identification of the site of MCL-1 modification using tandem mass spectrometry. **a** ESI-MS spectrum of irradiated mixture: 5 μM MCL-1, 10 μM Ac-RTA **4**,1 μM peptide **1**,10 μM APS, 1 min irradiation, 50 mM (NH_4_)HCO_3_, pH 7.4, showing unmodified MCL-1 (17737 Da) and modified MCL-1 (17914 Da). **b** Q-TOF MS/MS spectrum for a selected peptide modified with Ac-RTA **4**. Observed y and b ions are shown in red and blue, respectively. **c** Mapping of labelled cysteine residue Cys286 (and unmodified tyrosine residues Tyr175 and Tyr185) onto MCL-1/NOXA-B structure (PDB:2NLA). MCL-1 is shown in green; NOXA-B peptide is shown in cyan. The star at the *N*-terminus of the NOXA-B peptide denotes the position of attachment of linker and Ru(II)(bpy)_3_ complex.

Tandem mass spectrometry was used to identify the amino acid residue(s) on MCL-1 modified upon Ru(II)(bpy)_3_-NOXA-B **1**-mediated photolabelling. Minimal RTA **4** (Fig. 2a) was chosen over fluorescent and biotinylated RTAs **2** and **3**, due to the relative simplicity of the chemical structure, avoiding complication of MS/MS spectra due to fragmentation of the label in the mass spectrometer. For the photolabelling experiment, 10 μM RTA **4** and irradiation for 5 min were used to maximise conversion to the labelled species. Intact protein mass spectrometry (ESI-MS) of the labelling mixture prior to proteolytic digestion confirmed a high conversion in the labelling reaction, and incorporation of a single RTA label **4** (Fig. 3a), consistent with previous experiments with RTAs **2**-**4** described above.

Limited proteolysis of MCL-1 before and after modification with RTA **4**, using trypsin and Glu-C, resulted in peptide fragments that were analysed using reverse-phase HPLC and Q-TOF MS/MS (Supplementary Figs. 21 and 22; sequence coverage: 97% for unmodified MCL-1 and 94% for modified MCL-1). Peptide mapping analysis identified Cys286 as the only site on MCL-1 modified with label **4** (Fig. 3b, c, Supplementary Figs. 23 and 24), despite the presence of two tyrosine residues in the MCL-1 protein sequence (Tyr175 and Tyr185), which had been the expected sites for labelling based on previous reports on Ru (II)(bpy)_3_-based LDL reagents^33–36^. In addition, an MCL-1 Cys286Ser variant protein, which retains high affinity for BH3 peptides^52^, did not undergo Ru(II)(bpy)_3_-NOXA-B **1**-mediated photolabelling (Supplementary Fig. 25), which is consistent with the observed specificity of LDL with this particular protein-SET catalyst pair for Cys286 of MCL-1. The absence of any labelling on Tyr175 or Tyr185 may indicate that these residues are sterically restricted from reaction with the RTA.

### Exploring the ligand-directed nature of MCL-1 labelling

**Fig. 4:**
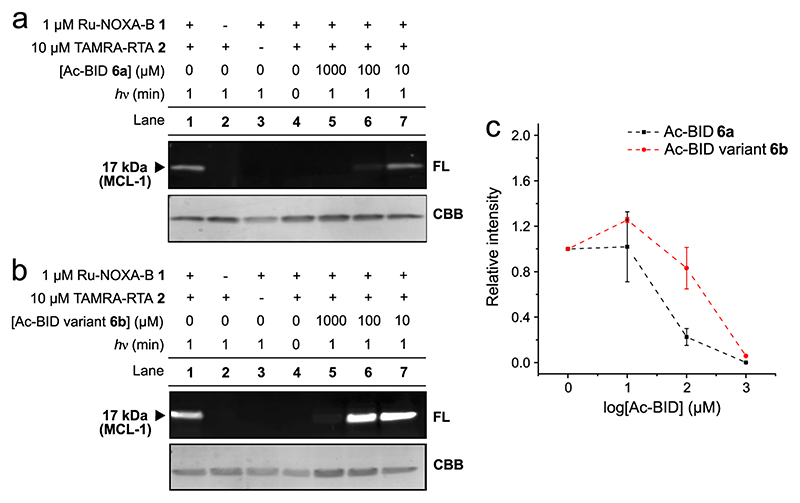
Competition experiments to test the ligand-directed nature of SET photolabelling. **a**, **b** SDS-PAGE gels showing that fluorescent labelling of MCL-1 mediated by peptide **1** is inhibited more efficiently by Ac-BID **6a** (a; lanes 4-7) than by Ac-BID variant **6b** (b; lanes 4-7). Conditions: 5 μM MCL-1,1 μM Ru-NOXA-B **1**,10 μM RTA **2**,10 μM APS, 0-1000 μM Ac-BID **6a** (in a) or Ac-BID variant **6b** (in b), 1 min irradiation, 50 mM (NH_4_)HCO_3_ (pH 7.4). Pictures of complete gels are shown in Supplementary Figs. 28 and 29. **c** Plot of relative fluorescence intensity (intensity of fluorescent band (FL)/intensity of Coomassie Brilliant Blue (CBB) band; normalised to the experiment in lane 1) for the competition experiments with different concentrations of Ac-BID **6a** and Ac-BID variant **6b**. Error bars represent the standard deviations from three independent repeats.

To test whether labelling of MCL-1 was indeed mediated by peptide binding, bringing the ruthenium complex into proximity of the amino acid(s) residue to be labelled, labelling experiments were also performed using non-targeted SET catalyst Ru(II)(bpy)_3_. MCL-1 could be labelled with Ac-RTA **4** in the presence of 20 mol% Ru(II)(bpy)_3_, but labelling was less efficient than with targeted SET catalyst Ru(II)(bpy)_3_-NOXA-B **1** (Supplementary Fig. 26a vs Supplementary Fig. 17a). Interestingly, MCL-1 Cys286Ser was not labelled in the presence of 20 mol% Ru(II) (bpy)_3_ (Supplementary Fig. 26b), reinforcing the notion that Cys286 is more susceptible to SET-mediated labelling than any other residue of MCL-1. Next, experiments were undertaken to test inhibition of Ru(II)(bpy)_3_-NOXA-B **1**-mediated MCL-1 labelling by competitor peptides. Initially, we explored the use of the wild-type sequence, Ac-NOXA-B **5**, did not completely abrogate labelling (Supplementary Fig. 27), presumably due to the 10-fold lower inhibitory potency of peptide **5** (2068 ± 334 nM, Fig. 2b) compared with ruthenium(II)-modified peptide **1** (201 ± 14 nM; Fig. 2b). Therefore, another peptide that binds in the same hydrophobic groove of MCL-1 as NOXA-B, but with a comparable IC_50_ value to that of **1** (Ac-BID **6a**, IC_50_ 390 ± 80 nM)^45^ was chosen. Increasing concentrations (0–1000 μM) of Ac-BID **6a** were added to the reaction mixture prior to irradiation, resulting in decreasing amounts of fluorescently modified protein. The intensity of the fluorescent and Coomassie-stained bands (Fig. 4a; Supplementary Fig. 28) was quantified using ImageJ software, and the data from three independent repeats were plotted against the concentration of competitor peptide **6a**, revealing a dosedependent inhibition of labelling—with labelling substantially inhibited (ca. 70% inhibition) using 100 μM Ac-BID **6a** and completely abolished using 1000 μM Ac-BID **6a** (Fig. 4c). In contrast, a variant Ac-BID sequence **6b**, with key hot-spot residues exchanged (L90A and D95R; Supplementary Figs. 7 and 8; Supplementary Table 3) resulting in loss of inhibitory potency against MCL-1 (IC_50_ ≫ 50 μM; Supplementary Fig. 13), was a less potent competitor of the photolabelling reaction, with minimal suppression of MCL-1 labelling observed at 100 μM of **6b** (Fig. 4b, c; Supplementary Fig. 29). The concentration of **6a** needed to suppress labelling may seem high compared to its IC_50_, but it should be noted that the labelling is a kinetically controlled, irreversible process, which will therefore depend on labelling time as well as relative affinities of peptide:MCL-1 complexes. In addition, it is possible that, in the presence of competitor peptide, Ru(II)(bpy)_3_-NOXA-B **1** acts as a non-targeted photocatalyst similar to Ru(II)(bpy)_3_; although this process would be less efficient (see above), it may not be inhibited efficiently by Ac-BID **6a**. It is noteworthy that 1000 μM Ac-BID variant **6b** almost fully suppressed MCL-1 photolabelling. Peptides **6a** and **6b** both incorporate a histidine and a tryptophan residue, millimolar concentrations of which have been shown to suppress Ru(II) (bpy)_3_-mediated protein crosslinking reactions in the presence of-APS^31^. Therefore, experiments with non-targeted SET reagent Ru (II)(bpy)_3_ (20 mol%) were also performed in the presence of Ac-BID **6a** (Supplementary Figs. 30 and 31). Minimal suppression of MCL-1 labelling (~20% inhibition) was seen with 100 μM Ac-BID **6a**, but at 1000 μM, Ac-BID **6a** almost completely suppressed Ru (II)(bpy)_3_-mediated labelling of MCL-1 (Supplementary Fig. 30b). Several explanations for these observations are possible. Because covalent labelling of Cys286 can lead to allosteric inhibition of MCL-1^53^, Ac-BID may modulate access to Cys286, the only site of MCL-1 labelled under the conditions used in these experiments (see above). Alternatively, Ac-BID **6a** may interact non-specifically with Ru(II)(bpy)_3_. However, at the highest concentration of Ac-BID **6a** (1000 μM), quenching of the SET reaction by its histidine and especially its tryptophan residue is likely to play a significant role. These combined results suggest that the specific binding between MCL-1 and Ru(II)(bpy)_3_-NOXA-B **1** is an important factor in the efficient SET photolabelling of MCL-1, and highlight potential limitations in the use of competition experiments to investigate the ligand-directed nature of SET photolabelling.

### MCL-1 is selectively labelled in mixtures of proteins

**Fig. 5:**
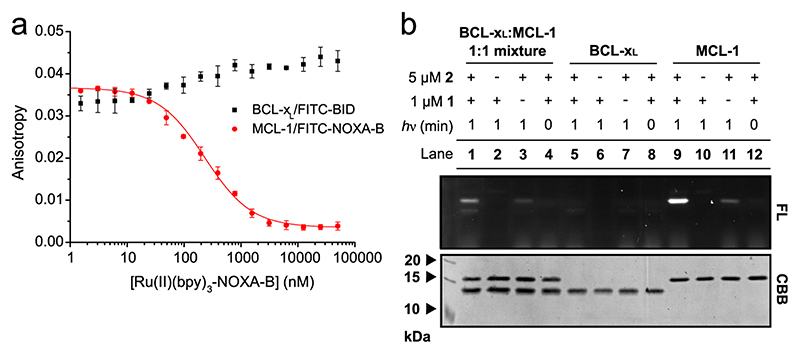
Selectivity of Ru(II)(bpy)_3_-NOXA-B 1 for MCL-1 over BCL-x_L_. **a** Fluorescence anisotropy competition experiment for the inhibition of the MCL-1/ FITC-NOXA-B interaction (red) and BCL-x_L_/FITC-BID interaction (black) by peptide **1**. Error bars represent the standard deviation of three repeats. **b** SDS-PAGE gel showing selective labelling of MCL-1 in a 1:1 mixture of MCL-1 and BCL-x_L_ (lane 1). BCL-x_L_ is not labelled in a solution of the protein on its own (lane 5). Conditions: 5 μM protein, 1 μM RTA **2**, 10 μM APS, 1 min irradiation, 50 mM (NH_4_)HCO_3_ (pH 7.4). Pictures of complete gels are shown in the Supplementary Fig. 33.

As an alternative approach to assessing the ligand-directed nature of SET photolabelling by Ru(II)(bpy)_3_-NOXA-B **1**, we determined whether Ru(II)(bpy)_3_-modified peptide **1** could selectively label MCL-1 over a structurally related BCL-2 family member to which **1** does not bind^45^. According to fluorescence anisotropy experiments, Ru(II)(bpy)_3_-NOXA-B **1** does not inhibit the interaction of BCL-x_L(1–198,Δ27–82)_^45^ with the fluorescently labelled peptide FITC-BID **7** (in comparison to an IC_50_ value of 201±14nM obtained for the MCL-1/FITC-NOXA-B interaction; Fig. 5a; Supplementary Fig. 12). Therefore, we evaluated the selectivity of ligand-directed photolabelling with **1** between MCL-1 and BCL-x_L_ (Fig. 5b; Supplementary Figs. 32 and 33). Indeed, in an equimolar mixture of MCL-1 and BCL-x_L_, MCL-1 was labelled selectively over BCL-x_L_ (lane 1, Fig. 5b). The experiments suggests that a trace amount of BCL-x_L_ can be labelled, but only when MCL-1 is present as well (compare lanes 1 and 5, Fig. 5b). Although there is tentative evidence for an interaction between MCL-1 and BCL-x_L_^54^, the observation that higher concentrations of TAMRA-RTA **2** (500 μM) led to more non-specific labelling of BCL-x_L_ (Supplementary Fig. 34) suggest this trace labelling results from the ability of TAMRA itself to act as non-targeted photocatalyst^46,47^. Indeed, when labelling was performed with Ac-RTA instead, ESI-MS analysis did not reveal any Ru(II) (bpy)_3_-NOXA-B **1**-mediated SET labelling (nor degradation) of BCL-x_L_ (Supplementary Fig. 35a). In contrast, small amounts of BCL-x_L_ could be labelled with Ac-RTA **4** when 20 mol% of nontargeted Ru(II)(bpy)_3_ was used instead, and the efficiency of BCL-x_L_ labelling (as well as its degradation) increased at higher concentrations of Ru(II)(bpy)_3_ (Supplementary Fig. 35b). MCL-1 was also selectively labelled in a stoichiometric mixture of MCL-1 and hDM2, a regulator of the p53 tumour suppressor (Supplementary Fig. 36). These results further confirm a ligand-directed mode of labelling, i.e., binding of Ru(II)(bpy)_3_-NOXA-B **1** to MCL-1 facilitates selective labelling of MCL-1 over proteins it does not bind to.

## Discussion

We have demonstrated the use of the peptide-based protein mimic Ru(II)(bpy)_3_-NOXA-B **1** as an LDL reagent for the selective photocatalytic, ligand-directed labelling of the anti-apoptotic BCL-2 protein, MCL-1. SET photolabelling of recombinant MCL-1 with three different dimethylaniline derivatives was achieved, with irradiation times of 1 min; in-gel fluorescence measurements and ESI-MS analysis allowed relative quantification of labelling. This work builds on previous literature describing the use of small molecule-based Ru(II)(bpy)_3_-based LDL reagents for the labelling of the enzymes CAII, EGFR and DHFR^33–35^. In contrast to previous reports^33,35,36^, a sub-stoichiometric amount of the Ru(II)(bpy)_3_ reagent (20 mol% relative to [MCL-1]) was used in these experiments, while larger amounts of **1** induced more degradation of MCL-1. This demonstrates the catalytic potential of the SET-based LDL technology as well as the suitability of peptide-based LDL reagents for application on interfaces of PPIs, and suggests that the balance between protein labelling and protein degradation needs to be considered carefully for each system. Collectively, experiments using targeted vs. non-targeted SET reagents, competition experiments, and labelling experiments in mixtures of proteins support a ligand-directed nature of SET photolabelling with Ru (II)(bpy)_3_-NOXA-B **1**. It should be noted that, due to the conserved sequence homology between BCL-2 family members, this network of PPIs presents a significant challenge for selective protein labelling.

Tandem MS experiments revealed that the predominant labelling site of MCL-1 with minimal dimethylaniline label **4** was a single cysteine residue (Cys286), an amino acid not previously reported to react via this type of LDL chemistry^37^. In addition, MCL-1 Cys286Ser was labelled by neither Ru(II)(bpy)_3_-NOXA-B **1** nor non-targeted Ru(II)(bpy)_3_, suggesting that, despite the presence of two tyrosine residues, Cys286 is the only MCL-1 residue able to undergo SET photolabelling under the conditions used in this study. Kodadek and co-workers reported that protein-protein crosslinking using Ru(II)(bpy)_3_ reagents can be inhibited by the addition of excess cysteine (or tyrosine, tryptophan, methionine or histidine)^31^, suggesting that thiols can trap or quench radicals formed upon photoexcitation. More importantly, Ru(II)(bpy)_3_ and related complexes have been used for the formation of thiol radicals and their use in C-S bond formation, including on peptides^55–57^. In addition, the group of Finn demonstrated the use of Ru(II)(bpy)_3_-mediated photolabelling of Tyr residues of viral capsid proteins with thiol derivatives^58^, suggesting that thiols can efficiently form C-S bonds with electron-rich aromatics in these types of reactions. It should be noted that the distance limits of SET-mediated protein labelling (and therefore residue selectivity) may depend on the contribution of different reaction pathways, which in turn may depend on the type of RTA used^36^. Further studies will be needed to unravel the precise mechanistic details of the labelling reaction between cysteine residues and dimethylaniline-based RTAs.

We found that the addition of APS as a co-oxidant was necessary for efficient protein labelling in vitro. However, previous literature describing intracellular protein labelling in the absence of APS proposes an alternative pathway whereby molecular oxygen acts as the electron acceptor and labelling is mediated by Ru (III) (which can be stabilised by consumption of superoxide, for example by addition of superoxide dismutase)^31,33^. Therefore, future studies will focus on the development of Ru(II)(bpy)_3_-based LDL to study PPIs in cells and/or cell lysates, which may ultimately enable identification of novel transient/weak PPIs, without the need of protein overexpression.

Chemical labelling approaches to study PPIs complement enzyme-mediated proximity labelling approaches such as BioID^59^ and APEX^60^. In comparison to traditional chemical crosslinking methods to study PPIs, photolabelling of proteins mediated by Ru (II)(bpy)_3_-modified peptides such as **1** presents a number of potential advantages. Compared with non-specific reagents such as DSSO, SDA and Sulfo-SBED, ligand-directed labelling of a protein of interest within a complex mixture may facilitate analysis of its individual interactome. Additionally, reagent **1** is unreactive in biological media, activated only upon irradiation at a specific time point, whereas electrophilic groups such as NHS esters or maleimides are susceptible to hydrolysis/reaction with bulk nucleophiles in biological environments. The sub-stoichiometric quantities of Ru(II)(bpy)_3_ peptide required for efficient labelling and short irradiation times (1 min) at longer wavelengths than UV-activated crosslinking reagents are also less likely to perturb the system under study. Based on reports by Kodadek et al.^31^ and on our recent experience with bespoke LEDbased irradiation systems for photoaffinity labelling^61^, we expect that labelling with shorter irradiation times can be achieved in future studies. This would allow future dynamic interactome studies with high temporal resolution.

## Methods

### Synthesis and characterisation

Full synthetic procedures are available in the Supplementary Methods.

### Fluorescence anisotropy

Assays were carried out in 384 well Optiplates and wells were read using a Perkin Elmer EnVision™ 2103 MultiLabel plate reader. Fluorescein-labelled peptides were examined using excitation and emission wavelengths of 480 nm and 535 nm, respectively (dichroic mirror 505 nm). All assays were performed in Tris buffer (50 mM Tris, 150 mM NaCl, 0.01% Triton X-100, pH 7.4). Direct titrations and competition assays were performed with minor modifications to those described previously^45^, and are detailed in full in the Supplementary Methods.

### General procedure for photolabelling of recombinant MCL-1

To a solution of MCL-1 (final concentration 5 μM) in ammonium acetate buffer (50 mM, pH 7.5) was added Ru(II)(bpy)_3_-NOXA-B (final concentration 1 μM), RTA (final concentration 5-50 μM) and APS (final concentration 10 μM), and the mixture was incubated at r.t. for 5 min. The mixture was irradiated for 1 min at r.t., 5 cm from the light source (Kessil H150W-BLUE LED, 32W, 2 × lamps) and then immediately quenched by the addition of DTT (final concentration 10 mM) and analysed using ESI-MS and/or SDS-PAGE. Fluorescently modified peptides were analysed using a Molecular Imager ChemiDoc XRS System (Bio-Rad, CA). The details of specific labelling experiments are provided in the Supplementary Methods.

### MS/MS identification of modified amino acid residue

To a solution of MCL-1 (5 μM) in ammonium acetate buffer (50mM, pH 7.5) was added Ru(II)(bpy)_3_-nOxA-B **1** (final concentration 1 μM), Ac-RTA **4** (final concentration 10 μM) and APS (final concentration 10 μM) and the mixture was incubated at r.t. for 5 min. The mixture was irradiated for 1 min at r.t., 5 cm from light source (Kessil H150W-BLUE LED, 32 W, 2 × lamps) and immediately quenched by the addition of DTT (final concentration 10 mM). The sample was split into two 50 μL aliquots. To each aliquot, a protease solution (Trypsin or Glu-C; Promega (Madison, WI); 20 ng μL^–1^ in 25 mM ammonium bicarbonate) was added in a 1:50 ratio (protease:total protein content). Samples were incubated at 37°C with shaking for 18h. The digest reaction was stopped by adding 5 μL of 1% HCOOH, then subjected to purification using a Sep-pak 18 column. The Sep-pak column was equilibrated with 1 mL 0.1% TFA. 500 μL of 0.1% TFA was added to the peptide digest, the mixture was passed through the column and the column was washed with 1 mL 0.1% TFA. Peptides were eluted from the column with 500 μL MeCN-H_2_O 1:1 + 0.1% HCOOH. The eluant was dried by vacuum centrifugation and the peptides were reconstituted in 20 μL 0.1% TFA. LC separation of the peptide mixtures was performed on an ACQUITY M-Class UPLC (Waters UK, Manchester). 1 μL sample was loaded onto a Symmetry C18 trap column and washed with 1% MeCN/0.1% HCOOH for 5 min at 5 μL min^-1^, then the peptides were separated on a HSS T3 C18 analytical column (Waters UK, Manchester) by gradient elution of 1-60% solvent B (0.1% HCOOH in MeCN) in A (0.1% HCOOH in H_2_O) over 30 min at 0.3 μL min^-1^. The peptides were analysed using a Xevo G2-XS Q-TOF mass spectrometer. Data processing was performed using the MassLynX v4.1 suite of software. Peptide MS/ MS data from both trypsin and Glu-C digests were processed with PEAKS Studio (Bioinformatics Solutions Inc., Waterloo, Ontario, Canada) and searched against the amino acid sequence. 176.0951 Da was set as a variable modification on any residues to determine the position of the Ac-RTA **4** modification. MS mass tolerance was 10 ppm, and fragment ion mass tolerance was 0.05 Da. The false discovery rate was set to 1%.

## Supplementary Material

**Supplementary information** is available for this paper at https://doi.org/10.1038/s42004-019-0235-z.

SuppMethods

## Figures and Tables

**Fig. 1:**
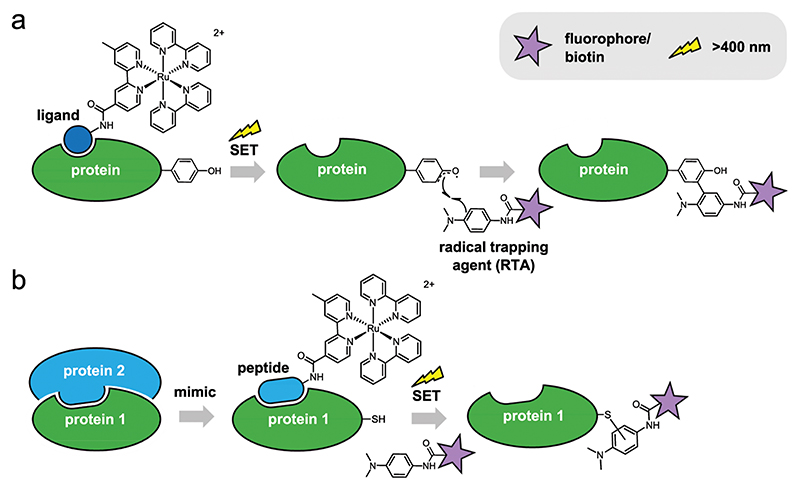
Ligand-directed protein labelling using catalytic Ru(II)(bpy)_3_ reagents. **a** Previous work: use of small-molecule ligands to promote selective target protein labelling *via* generation and trapping of tyrosyl radicals. **b** This work: use of a NOXA-B BH3 peptide as the recognition element (ligand), mimicking an interacting partner within the MCL-1/NOXA-B PPI and facilitating SET-mediated ligand-directed MCL-1 labelling.

## Data Availability

The authors declare that the data supporting the findings of this study are available within the article and Supplementary Information file, or from the corresponding authors upon reasonable request.
