# Supplementary material for: Photocatalytic proximity labelling of MCL-1 by a BH3 ligand
Source: Commun Chem. Author manuscript; Available in PMC 2021 Mar 23. (PMC7610391; doi:10.1038/s42004-019-0235-z)
Supplement: SuppMethods [file EMS117797-supplement-SuppMethods.pdf]

Supplementary Methods

General comments

All chemical reagents were purchased from commercial suppliers and used without further purification. When used as reaction solvents, THF and CH_2Cl_2 were dried and deoxygenated using an Innovative Technology Inc. PureSolv® solvent purification system.

Flash column chromatography was carried out using silica (Merck Geduran silica gel, 35–70 μm particles) according to the method described by Still, Kahn and Mitra.[1] Thin layer chromatography was carried out on commercially available pre-coated aluminium plates (Merck silica 2 8 8 0 Kieselgel 60 F_{254}). Analytical HPLC (small molecules) was performed on an Agilent 1290 Infinity Series equipped with a UV detector and a Hyperclone C_{18} reverse phase column using MeCN/water (5→95%) containing 0.1% formic acid, at either 0.5 mL min^{-1} over a period of five minutes or 1.0 mL min^{-1} over a period of 30 minutes.

High resolution electrospray (ESI+) mass spectrometry was performed on a Bruker MaXis Impact QTOF mass spectrometer, and *m/z* values are reported in Daltons to four decimal places. LC-ESI-MS data were recorded on an Agilent Technologies 1200 series HPLC combined with a Bruker HCT Ultra ion trap using 50 × 20 mm C_{18} reverse phase columns using MeCN/water (5→95%) containing 0.1% formic acid. A flow rate of 1.5 mL min^{-1} was used and *m/z* values are given in Daltons to one decimal place.

^{1}H and ^{13}C NMR spectra were recorded in deuterated solvents on a Bruker Avance 500 or Bruker Avance DPX 300. Chemical shifts are quoted in parts per million downfield of tetramethylsilane and referenced to residual solvent peaks ($CDCl_3$: ^{1}H = 7.26 ppm, ^{13}C = 77.16 ppm, DMSO-d_6: ^{1}H = 2.50 ppm, ^{13}C = 39.52 ppm) and coupling constants (*J*) are reported to the nearest 0.1 Hz. Assignment of spectra was based on expected chemical shifts and coupling constants, aided by COSY, HSQC and HMBC measurements where appropriate.

Fourier-transform infrared absorption spectroscopy (IR) was performed on Bruker Platinum-ATR system equipped with an Alpha FT-IR spectrometer. Maximum absorbances are reported for significant bands in cm^{-1}.

Synthesis of Ru(II)(bpy)$_3$ complex for peptide capping

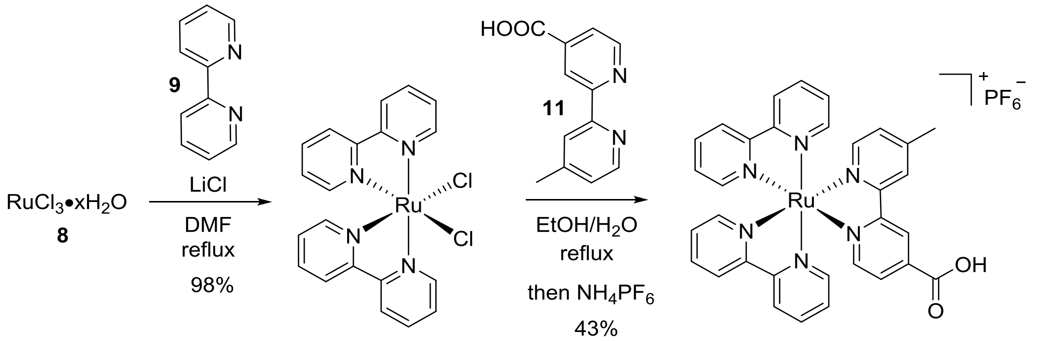

***Supplementary Figure 1:** Synthetic route to heteroleptic ruthenium(II) bipyridine complex **12** (prepared according to the previously reported method by Meyer et al.)*[2]

Tris(2,2'-bipyridine)ruthenium(II) dichloride 10[3]

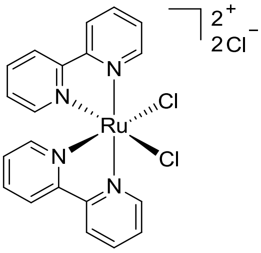

To a solution of ruthenium(III) chloride hydrate (500 mg, 2.41 mmol) in DMF (7.5 mL) was added lithium chloride (717 mg, 16.90 mmol), followed by 2,2'-bipyridine (753 mg, 4.82 mmol) in three equal portions, and the reaction mixture was heated at reflux overnight. The reaction mixture was cooled to room temperature, acetone was added (40 mL) and the mixture was left at -4°C overnight. The brown microcrystals were filtered, washed with Et_2O (30 mL) and water (10 mL) to afford a brown solid (1.15 g, 98%). The crude product was used without further purification. HRMS (ESI): calcd. for $C_{20}H_{16}Cl_2N_4NaRu$ $[M+Na]^+$ 506.9686, found 506.9685. Data consistent with literature.

Bis-(2,2'-bipyridine)-(4'-methyl-[2,2'-bipyridine]-4-carboxylic acid)ruthenium (II) bis-(hexafluorophosphate) 12[2]

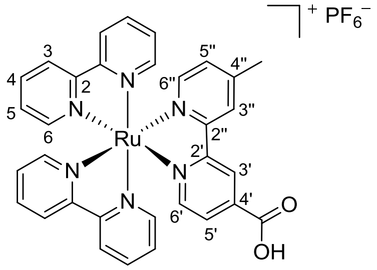

To a solution of crude **10** (905 mg, 1.87 mmol) in a mixture of EtOH (42 mL) and water (18 mL), was added 4-methyl-2,2'-bipyridine-4'-carboxylic acid (250 mg, 1.17 mmol), and the

reaction mixture was heated at reflux for 24 hours. The reaction mixture was cooled to room temperature, the solvents were removed *in vacuo* and the residue was re-suspended in water (30 mL) and filtered. The crude product was purified by FCC (SiO_2, eluting with 100:10:1→100:20:1, v/v/v, MeCN:H_2O:KNO_3(sat.)), solvents were removed *in vacuo*, and the residue was re-dissolved in water (30 mL). Ammonium hexafluorophosphate (500 mg) was added in portions, resulting in the formation of a red-orange precipitate, which was collected *via* vacuum filtration and washed with H_2O (20 mL) to give the product as dark red crystals (740 mg, 43%). R_f 0.15 (100:20:1, v/v/v, MeCN:H_2O:KNO_3(sat.)); δ_H (500 MHz, acetonitrile-d_3) 8.93 (1H, s, 3'-C*H*), 8.53 – 8.45 (5H, m, 3-C*H,* 3''-C*H*), 8.05 (4H, m, 6-C*H*), 7.82 – 7.69 (6H, m, 4-C*H,* 6'-C*H,* 6''-C*H*), 7.54 (1H, d, *J* 5.8, 5'-C*H*), 7.44 – 7.34 (4H, m, 5-C*H*), 7.24 (1H, d, *J* 5.5, 5''-C*H*), 2.53 (3H, s, C*H*$_3$); δ_C (125 MHz, acetonitrile-d_3) 167.6 (*C*(O)OH), 158.1 – 157.9 (*C*-2, *C*-2', *C*-2''), 152.8 (*C*H-6'), 152.7 (*C*H-4), 152.6 (*C*-4'), 151.65 (*C*H-5''), 146.8 (*C*-4''), 138.7 (*C*H-6), 129.4 (*C*H-5''), 128.6 (*C*H-5), 127.7 (*C*H-6''), 126.3 (*C*H-3''), 125.2 (*C*H-3), 124.3 (*C*H-3'), 21.15 (*C*H$_3$); IR (neat, ν_{max}/cm^{-1}) 3320, 1726, 1618, 1424, 1233, 825; HRMS (ESI): calcd. for $C_{32}H_{26}N_6O_2{}^{102}Ru$ $[M]^{2+}$ 315.0581, found 315.0583. Data consistent with literature.

Synthesis of peptides – general comments

All amino acids and resins were purchased from either Novabiochem (Merck) or Sigma-Aldrich. All amino acids were *N*-Fmoc protected and side chains protected with Boc (Lys); ^{t}Bu (Asp, Glu, Ser, Thr, Tyr); Trt (Asn, Gln); Pbf (Arg). Synthesis of all peptides was performed using a microwave assisted automated peptide synthesiser (CEM, Liberty Blue). Peptide acetylation was performed manually. DMF used in peptide synthesis was of HPLC grade and from Sigma Aldrich. Peptides were synthesised on a 0.125 mmol scale. Lyophilisation was performed using a BenchTop Pro with Omnitronics™ (VirTis SP Scientific). Preparative HPLC was performed on an Agilent Technologies 1260 infinity controller in conjunction with a diode array detector. Analytical HPLC experiments were performed on an Agilent Technologies 1260 infinity controller in conjunction with a diode array detector. Mass spectrometry data were obtained on a Bruker Daltonics microTOF using electrospray ionisation (ESI) MS instruments as appropriate. FITC-NOXA-B (FITC-Ahx-AAQLRRIGDKVNLRQKLLN-NH_2), FITC-BID (FITC-Ahx-E^{80}DIIRNIARHLAQVGDSN$_L$DRSIW-NH_2) and Ac-BID (Ac-E^{80}DIIRNIARHLAQVGDSN$_L$DRSIW-NH_2) were prepared previously.[4,5]

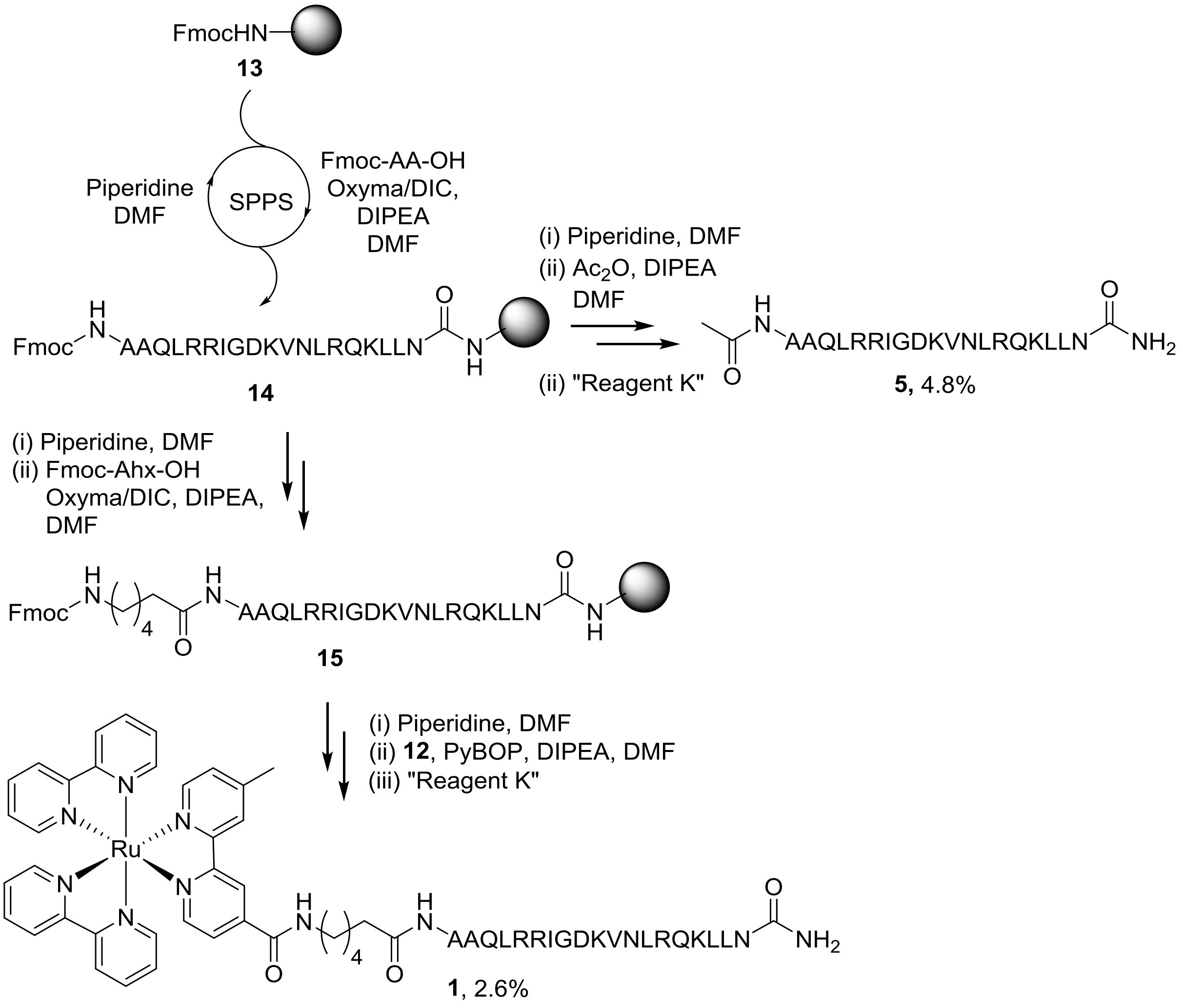

Supplementary Figure 2: *Synthetic route to acetylated and Ru(II)(bpy)$_3$ derivatives of NOXA-B peptide. Cleavage cocktail "Reagent K" is composed of TFA:EDT:thioanisole:phenol:H_2O, 82:3:5:5:5.*

Procedure for automated SPPS

Resin Loading: Clean reaction vessel; wash with DMF; transfer resin to reaction vessel; wash with DMF:CH_2Cl_2 (1:1); vessel draining.

Deprotection and Coupling: Add 20% piperidine in DMF (4 mL); microwave method (30 sec); wash with DMF (4 × 4 mL); drain; add amino acid (2.5 mL); add coupling reagent (1 mL); add base (0.5 mL); microwave method (1 min); wash through manifold to waste (2 mL); drain. For the deprotection and coupling of methods that did not use microwave assistance, the reactions were performed by agitation of the resin at r.t. for 10 min and 90 min, respectively. After the final residue, the resin was ejected from the reaction vessel and cleavage and deprotection was performed manually using methods A to D (see below).

Method A: Deprotection of N-Fmoc protecting groups

N-terminal Fmoc protecting groups were removed by the addition of 20% piperidine: DMF (5 × 2 mL × 2 min), followed by rinsing the resin with DMF (5 × 2 mL × 2 min). Successful deprotection was determined by a positive colour test (Method B).

Method B: Kaiser Test

The Kaiser Test was used to determine successful coupling or deprotection of manually coupled residues. A few beads of resin were placed in a vial, two drops of each solution (1-3, see below) was added to the beads and the solution was heated to ca. 100 °C for 1 minute. Successful coupling was indicated by no change in colour of the beads, whereas successful deprotection was indicated by bright blue beads.

1) Ninhydrin (5% w/v) in ethanol
2) Phenol (80% w/v) in ethanol
3) 1 mM KCN $_{(aq.)}$ in pyridine (2% v/v)

Method C: N-terminal acetylation

Acetic anhydride (10 equiv.) and DIPEA (10 equiv.) were dissolved in DMF (1 mL) and the solution was transferred to the resin. After 2 h, the resin was drained, washed with DMF (3 × 2 mL × 2 min) and successful capping determined by a negative colour test (Method B).

Method D: Cleavage and deprotection of Rink amide MBHA resin

After elongation and *N*-terminal capping was complete, the resin was washed with DMF (5 × 2 mL × 2 min), CH_2Cl_2 (5 × 2 mL × 2 min), Et_2O (5 × 2 mL × 2 min) and dried under vacuum for 1 h. Peptides were simultaneously cleaved and side-chain deprotected using 'Reagent K'

TFA:EDT:thioanisole:phenol:H_2O, 82:3:5:5:5 (1 × 2 mL × 2 h). The solution was precipitated in ice-cold Et_2O (50 mL), placed in a centrifuge (3000 rpm × 1 min) and the supernatant was removed. The precipitate was washed with ice-cold Et_2O (3 × 30 mL) and the washed precipitate was dried under a stream of nitrogen (1 h), before being dissolved in H_2O and lyophilised.

Peptide purification

Peptides were purified by preparative mass-directed HPLC using a Jupiter Proteo C_{18} preparative column on an increasing gradient of acetonitrile in water + 0.1% HCOOH (v/v) at a flow rate of 10 mL min^{-1}. Crude peptides were suspended in acetonitrile at an approximate concentration of 20 mg mL^{-1}. Purification runs injected a maximum of 0.4 mL of crude peptide solution and were allowed to run for 30 min, with acetonitrile increasing at a stated gradient. The mass directed chromatography software Masshunter by ChemStation (Agilent) was used to allow the collection of the desired peptide by mass, with the eluent split into an Agilent 6120 Quadropole LC-MS which triggers collection of eluent at a programmed m/z. Fractions containing purified peptide were combined, concentrated *in vacuo* and lyophilised.

Synthesis of Ru(II)(bpy)$_3$-NOXA-B peptide 1

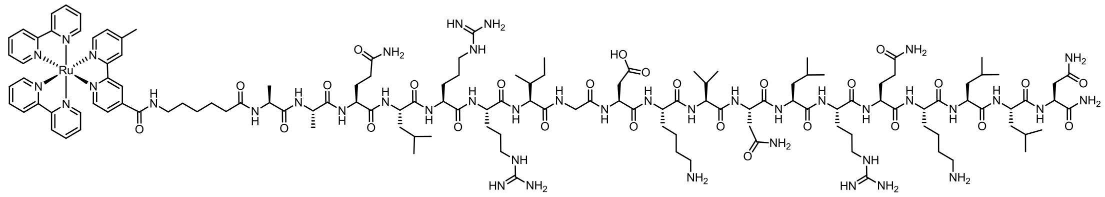

Ru(II)(bpy)$_3$-Ahx-AAQLRRIGDKVNLRQKLLN-NH$_2$ **1**

Ru(II)(bpy)$_3$-NOXA-B was synthesised on a 0.125 mmol scale on rink amide MBHA LL resin (0.35 mmol g^{-1}) using procedures described above all residues following isoleucine were double coupled. The *N*-terminus was elongated by dissolving Fmoc-6-aminohexanoic acid (5 equiv.), HCTU (5 equiv.) and DIPEA (5 equiv.) in DMF (2 mL) and the mixture was added to the resin, followed by agitation for 2 h. After removal of the reagents by filtration, the resin was washed with DMF (3 × 2 mL × 2 min) and the success of coupling determined by a negative colour test (Method B). Deprotection of the Fmoc-protected *N*-terminus then followed (Method A). The *N*-terminus was elongated by dissolving Ru(II)(bpy)$_3$PF$_6$ **12** (1.5 equiv.), PyBOP (3 equiv.) and DIPEA (5 equiv.) in DMF (2 mL) and the mixture was added to the resin, followed by agitation for 2 h (double coupling). After removal of the reagents by filtration, the resin was washed with DMF (3 × 2 mL × 2 min) and the success of coupling determined by a negative colour test (Method B). The peptide was simultaneously cleaved and side-chain deprotected using Method D. Ru(II)(bpy)$_3$-NOXA-B **1** was purified using mass-directed HPLC

over a gradient of 15-25% acetonitrile in H_2O + 0.1% HCOOH, to give (3.9 mg, 1.1%) of the final peptide.

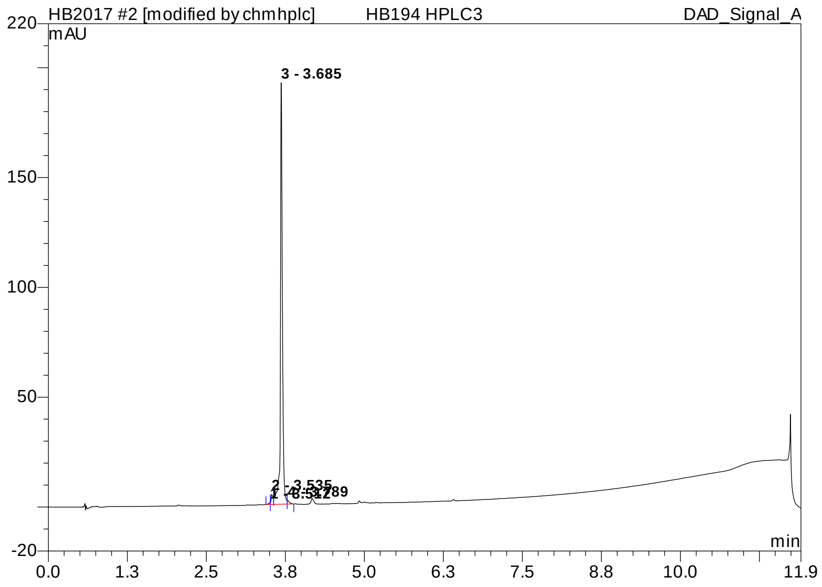

Supplementary Figure 3: *Analytical HPLC data for Ru(II)(bpy)$_3$-NOXA-B peptide* ***1***.

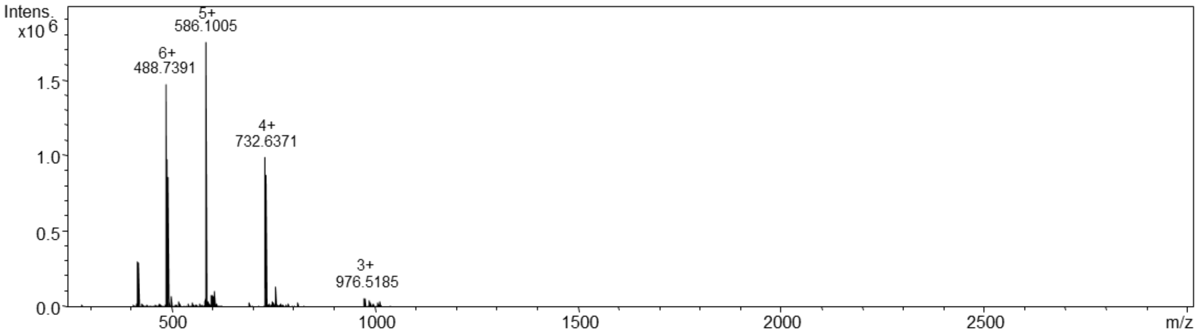

Supplementary Figure 4: *HRMS (ESI) spectrum for Ru(II)(bpy)$_3$-NOXA-B peptide* ***1****.*

Supplementary Table 1: *Tabulated HRMS data for Ru(II)(bpy)$_3$-NOXA-B peptide* ***1*** *showing the expected (Exp^d) and observed (Obs^d) masses for the multiple charge states.*

**Peptide**	**$[M+3H]^{3+}$ Obs^d**	**$[M+3H]^{3+}$ Exp^d**	**$[M+4H]^{4+}$ Obs^d**	**$[M+4H]^{4+}$ Exp^d**
Ru(II)bpy)$_3$-NOXA-B	976.5187	976.5162	732.6371	732.6391

Synthesis of Ac-NOXA-B 5

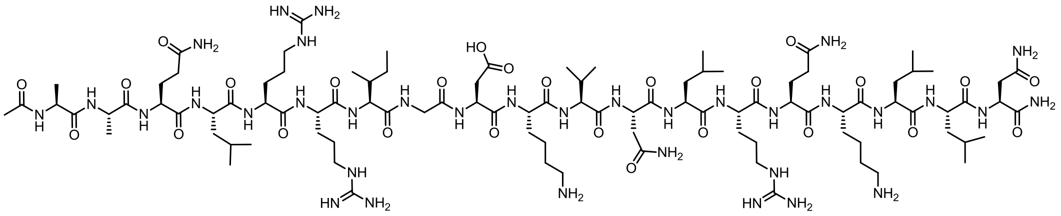

Ac-AAQLRRIGDKVNLRQKLLN-NH_2 **5**

Ac-NOXA-B **5** was synthesised on a 0.125 mmol scale on rink amide MBHA LL resin (0.35 mmol g^{-1}) using procedures described above; all residues following isoleucine were double coupled. The *N*-terminus was acetylated as described above (Method C) and the peptide was simultaneously cleaved and side-chain deprotected using Method D. Ac-NOXA-B **5** was purified using mass-directed HPLC over a gradient of 5-50% acetonitrile in H_2O + 0.1% HCOOH, to give (4.1 mg, 1.5%) of the final peptide.

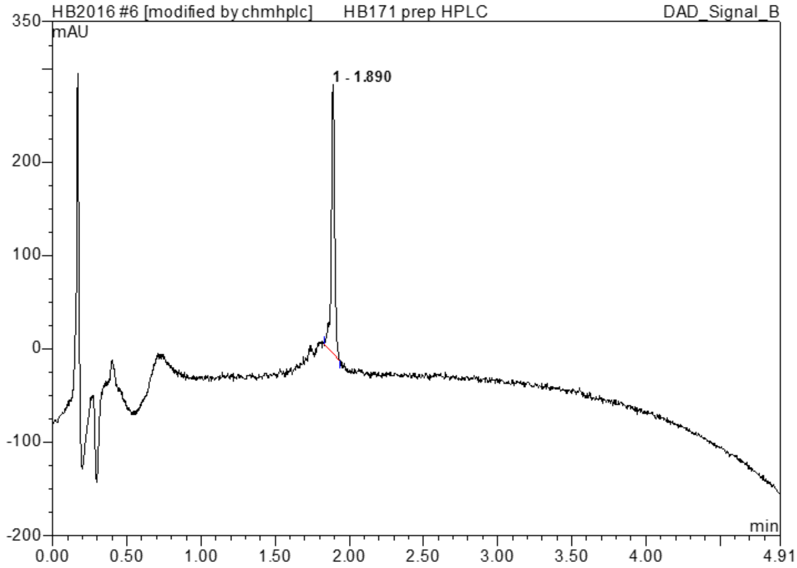

Supplementary Figure 5: *Analytical HPLC data for Ac-NOXA-B peptide* ***5***.

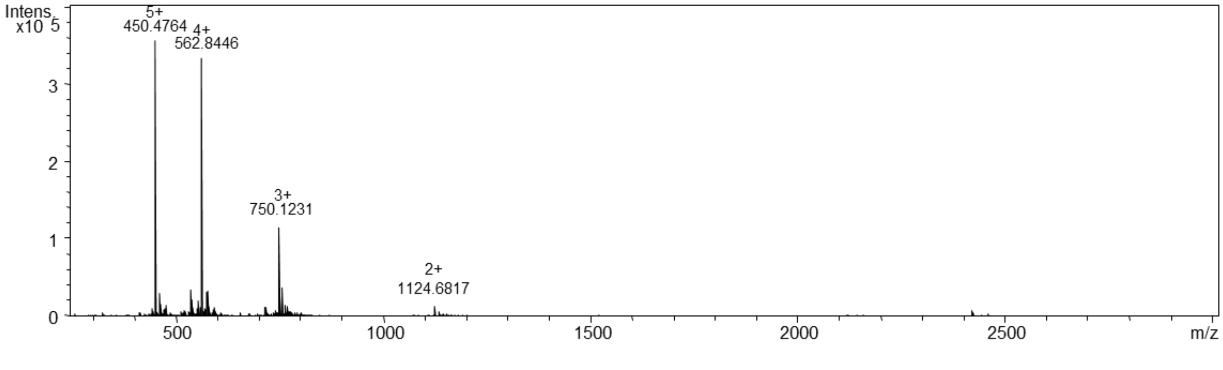

Supplementary Figure 6: *HRMS (ESI) spectrum for Ac-NOXA-B peptide* ***5***.

Supplementary Table 2: *Tabulated HRMS data for Ac-NOXA-B peptide **5** showing the expected (Exp[d]) and observed (Obs[d]) masses for the multiple charge states.*

Peptide	$[M+3H]^{3+}$ Obs[d]	$[M+3H]^{3+}$ Exp[d]	$[M+4H]^{4+}$ Obs[d]	$[M+4H]^{4+}$ Exp[d]
wt NOXA-B	750.1231	749.7889	562.8446	562.2762

Synthesis of Ac-BID variant 6b

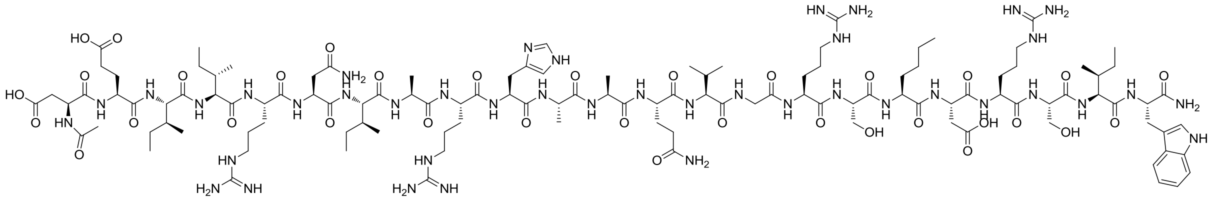

Ac-EDIIRNIARHAAQVGRS(Nle)DRSIW-NH_2 **6b**

Ac-BID variant **6b** was synthesised on a 0.1 mmol scale on rink amide MBHA LL resin (0.35 mmol g^{-1}) using procedures described above; The *N*-terminus was acetylated as described above (Method C) and the peptide was simultaneously cleaved and side-chain deprotected using Method D. Ac-BID variant **6b** was purified using mass-directed HPLC over a gradient of 5-50% acetonitrile in H_2O + 0.1% HCOOH, to give (21.4 mg, 7.9%) of the final peptide.

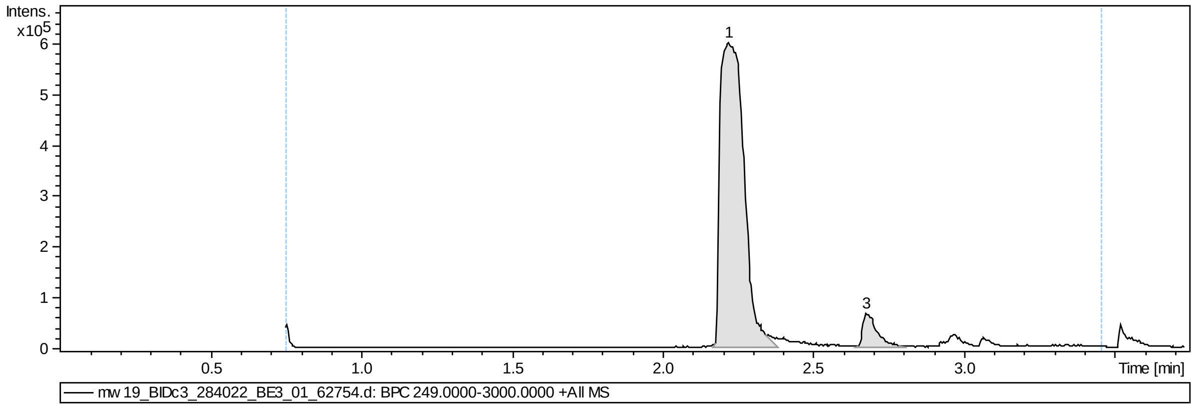

Supplementary Figure 7: *ESI-LC-MS data for Ac-BID variant **6b**.*

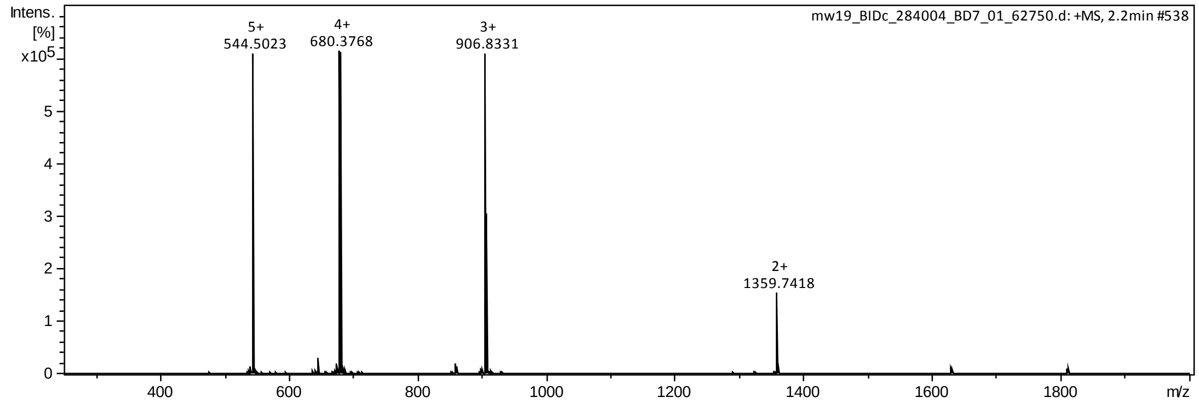

Supplementary Figure 8: *HRMS (ESI) spectrum for Ac-BID variant* ***6b****.*

Supplementary Table 3: *Tabulated HRMS data for Ac-BID variant* ***6b*** *showing the expected (Exp^{d}) and observed (Obs^{d}) masses for the multiple charge states.*

Peptide	$[M+3H]^{3+}$ Obs^{d}	$[M+3H]^{3+}$ Exp^{d}	$[M+4H]^{4+}$ Obs^{d}	$[M+4H]^{4+}$ Exp^{d}
Ac-BID variant **6b**	906.8331	906.8237	680.3768	680.3698

Synthesis of Radical Trapping Agents (RTAs)

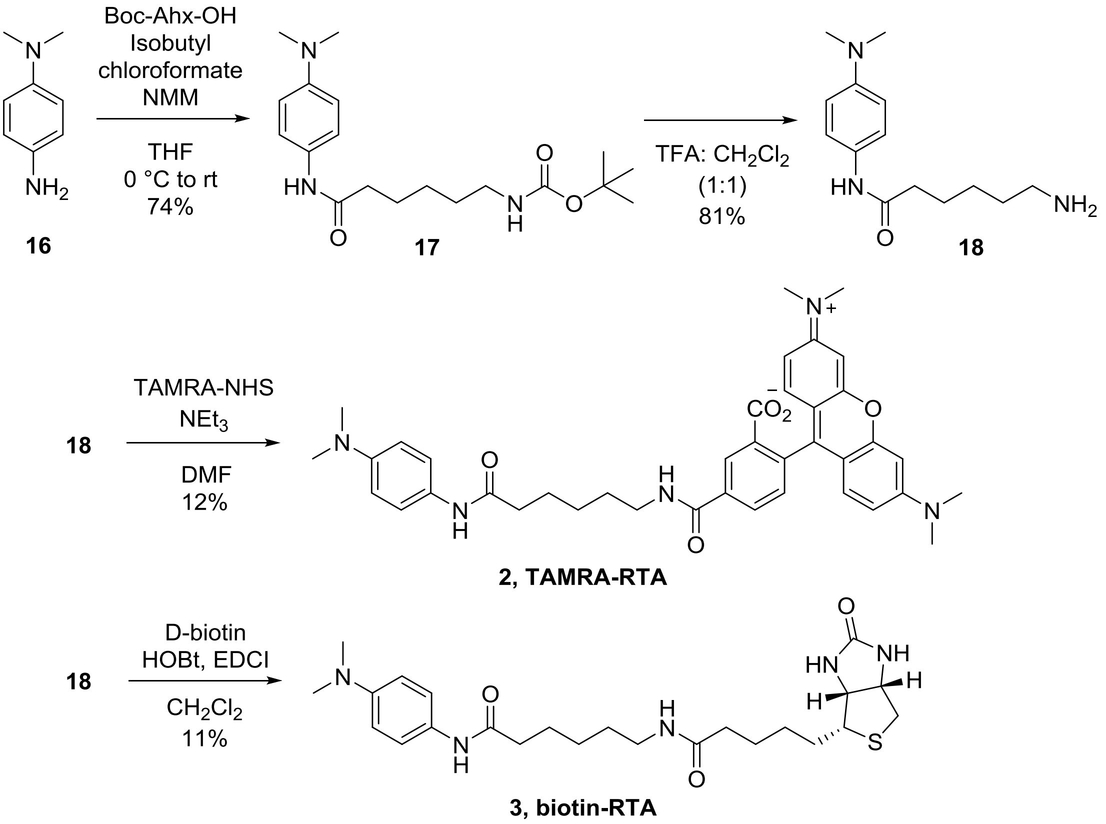

Supplementary Figure 9: *Synthetic routes to radical trapping agents **2** and **3**. Both fluorescent (**2**, TAMRA-RTA) and biotinylated (**3**, biotin-RTA) labels were synthesised from common building block **18**, in a divergent manner.*

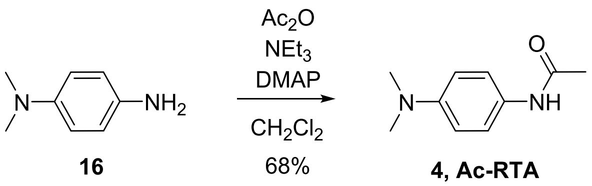

Supplementary Figure 10: *Synthesis of minimal RTA **4**, via acetylation of N,N-dimethyl-p-phenylenediamine **16**.*

***Tert*-butyl (6-((4-(dimethylamino)phenyl)amino)-6-oxohexyl)carbamate 17**

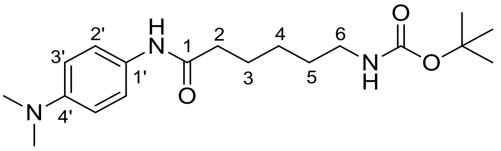

To a solution of Boc-Ahx-OH (500 mg, 2.16 mmol) in THF (8 mL) at 0 °C (ice-bath) was added isobutyl chloroformate (305 µL, 2.36 mmol) slowly, then *N*-methylmorpholine (402 µL, 3.65 mmol) slowly. The resulting solution was stirred for 30 min at 0 °C, then allowed to warm to

ambient temperature. After 1 h, a solution of *N,N*-dimethyl-*p*-phenylenediamine (478 mg, 3.51 mmol) in THF (0.5 mL) was added (solution turned dark green/brown) and the reaction mixture was stirred at ambient temperature overnight. The reaction mixture was quenched with 1 N HCl (ca. 5 mL) and washed with EtOAc (3 × 3 mL). A solution of 6 N NaOH was added to the mixture to become basic (pH 8). The product was extracted with EtOAc (5 × 10 mL), the organic layers were dried (Na_2SO_4), filtered and concentrated *in vacuo*. The crude product was purified by FCC (SiO_2, Biotage, 40 g, eluting with 12-70% EtOAc-Hex) to give the *title compound* as a purple solid (558 mg, 74%). R_f 0.31 (1:1 EtOAc-Hex); δ_H (400 MHz, $CDCl_3$) 7.43 (1H, s, N*H*), 7.38 – 7.32 (2H, app. d, *J* 9.0, 2'-C*H*), 6.74 – 6.62 (2H, app. d, *J* 9.0, 3'-C*H*), 4.70 – 4.55 (1H, s, N*H*), 3.09 (2H, m, 6-CH_2), 2.89 (6H, s, N-CH_3), 2.30 (2H, t, *J* 7.5, 2-CH_2), 1.71 (2H, p, *J* 7.5, 3-CH_2), 1.54 – 1.30 (13H, m, O^{t}Bu-CH_3, 4-CH_2, 5-CH_2); δ_C (100 MHz, $CDCl_3$) 171.1 (*C*-1), 156.2 (*C*(O)-^{t}Bu), 148.0 (*C*-4'), 128.2 (*C*-1'), 121.9 (*C*H-2'), 113.3 (*C*H-3'), 79.2 (*C*-^{t}Bu), 41.1 (CH_3-N), 40.5 (CH_2-6), 37.4 (CH_2-2), 29.9 (CH_2-3), 28.6 (CH_3-^{t}Bu), 26.5 (CH_2-5), 25.4 (CH_2-4); IR (neat, ν_{max}/cm^{-1}) 3373, 2932, 1649, 1513, 1159; HRMS (ESI): calcd. for $C_{19}H_{31}N_3NaO_3$ $[M+Na]^+$ 372.2258, found 372.2257.

6-amino-*N*-(4-(dimethylamino)phenyl)hexanamide 18

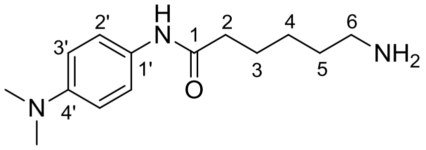

To a solution of **17** (1 g, 2.87 mmol) in CH_2Cl_2 (10 mL) was added trifluoroacetic acid (10 mL) and the reaction mixture was stirred at ambient temperature for 1.5 hours. The solvents were removed *in vacuo* to give a purple oil. The crude product was purified by FCC (SiO_2, eluting with CH_2Cl_2-MeOH-NEt_3 95:4:1→90:9:1) to afford the *title compound* as a pale purple waxy solid (681 mg, 95%). R_f 0.08 (CH_2Cl_2-MeOH-NEt_3 90:9:1); δ_H (400 MHz, $CDCl_3$) 7.70 (1H, s, N*H*), 7.41 – 7.30 (2H, m, 2'-C*H*), 6.74 – 6.62 (2H, app. d, *J* 9.0, 3'-C*H*), 2.88 (6H, m, N-CH_3), 2.68 (2H, t, *J* 6.9, 6-CH_2), 2.30 (4H, m, 2-CH_2, NH_2), 1.70 (2H, p, *J* 7.5, 3-CH_2), 1.55 – 1.30 (4H, m, 4-CH_2, 5-CH_2); δ_C (100 MHz, $CDCl_3$) 171.4 (*C*-1), 148.0 (*C*-4'), 128.2 (*C*-1'), 121.9 (*C*H-2'), 113.2 (*C*H-3'), 41.8, (*C*H_2-6), 41.1 (*C*H_3-N), 37.4 (*C*H_2-2), 32.9 (*C*H_2-5), 26.5 (*C*H_2-4), 25.6 (*C*H_2-3); IR (neat, ν_{max}/cm^{-1}) 3282, 2930, 1644, 1517, 1315; HRMS (ESI): calcd. for $C_{14}H_{24}N_3O$ $[M+H]^+$ 250.1914, found 250.1912.

2-(6-(dimethylamino)-3-(dimethyliminio)-3*H*-xanthen-9-yl)-5-((6-((4-dimethylamino) phenyl)amino)-6-oxohexyl)carbamoyl)benzoate (TAMRA-RTA) 2

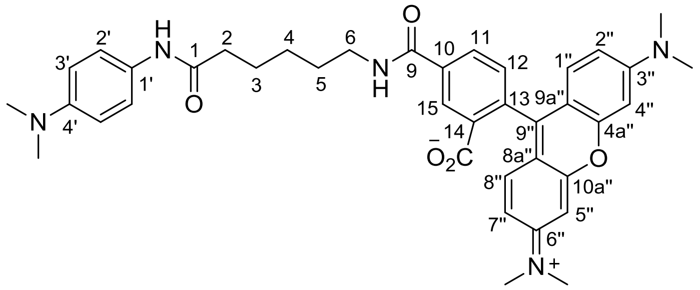

To a solution of amine **18** (50 mg, 0.20 mmol) in DMF (1 mL) was added TAMRA-NHS (138 mg, 0.26 mmol) and NEt_3 (56 µL, 0.40 mmol) and the mixture was stirred at ambient temperature for 20 hours. The solvent was removed *in vacuo* and the residue was re-dissolved in $CHCl_3$ (5 mL). The organic layer was washed with water (1 × 2 mL), saturated aqueous $NaHCO_3$ (aq., 3 × 2 mL), brine (1 × 2 mL), dried over anhydrous Na_2SO_4, filtered and concentrated to give a purple solid. The crude product was purified by FCC (SiO_2, eluting with CH_2Cl_2-MeOH 19:1→9:1) to afford the title compound as a purple solid (11 mg, 12%). R_f 0.12 (CH_2Cl_2-MeOH 9:1); δ_H (500 MHz, $CDCl_3$) 8.43 (1H, s, 15-C*H*), 8.08 (1H, d, *J* 8.2, 11-C*H*), 7.37 – 7.30 (2H, m, 2'-C*H*), 7.14 (1H, d, *J* 7.9, 12-C*H*), 6.90 (2H, d, *J* 8.9, 1''-C*H*, 8''-C*H*), 6.66 – 6.60 (2H, m, 3'-C*H*), 6.61 – 6.46 (5H, m, 2''-C*H*, 4''-C*H*, 5''-C*H*, 7''-CH, N*H*), 3.42 (2H, t, *J* 6.8, 6-CH_2), 3.02 (6H, s, rhodamine-N-CH_3), 2.89 (6H, s, rhodamine-N CH_3), 2.82 (6H, s, N-CH_3), 2.28 (2H, t, *J* 7.5, 2-CH_2), 1.68 (4H, m, 3-CH_2, 5-CH_2), 1.44 (2H, p, *J* 7.7, 4-CH_2); IR

(neat, v_{max}/cm^{-1}) 3496, 2998, 2853, 1651, 1493, 1320, 1058; HRMS (ESI): calcd. for $C_{39}H_{45}N_5O_5$ $[M+2H]^{2+}$ 331.6705, found 331.6705.

***N*-(4-(dimethylamino)phenyl)-6-(5-((4*S*)-2-oxohexahydro-1*H*-thienol[3,4-*d*]imidazol-4-yl)pentanamido)hexanamide (biotin-RTA) 3**

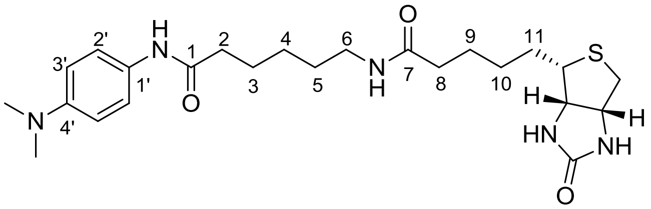

To a solution of D-biotin (421 mg, 1.72 mmol) in $CHCl_3$ (20 mL) was added EDCI (331 mg, 1.72 mmol), HOBt (233 mg, 1.72 mmol), amine **18** (286 mg, 1.15 mmol) and DIPEA (300 µL, 1.72 mmol), and the reaction mixture was stirred at ambient temperature overnight. The solvents were removed *in vacuo* to give a pale purple solid. The crude product was purified by FCC (SiO_2, eluting with CH_2Cl_2-MeOH 19:1→9:1) to afford the *title compound* as a colourless solid (182 mg, 33%). R_f 0.32 (CH_2Cl_2-MeOH 9:1); δ_H (400 MHz, $CDCl_3$) 7.56 – 7.39 (2H, app. d, *J* 9.0, 2'-C*H*), 6.85 (2H, app. d, *J* 8.9, 3'-C*H*), 4.59 (1H, dd, *J* 7.9, 4.8, biotin-C*H*), 4.39 (1H, dd, *J* 7.9, 4.4, biotin-C*H*), 3.48 (1H, s, biotin-C*H*), 3.28 (3H, m, 6-CH_2, biotin-C*H*), 3.16 – 2.86 (6H, m, N-CH_3), 2.82 (1H, d, *J* 12.8, biotin-C*H*), 2.45 (2H, t, *J* 7.4, 2-CH_2), 2.29 (2H, t, *J* 7.1, 8-CH_2), 1.90 – 1.59 (8H, m, 3-CH_2, 9-CH_2, 10-CH_2, 11-CH_2), 1.48 – 1.34 (4H, m, 4-CH_2, 5-CH_2); δ_C (100 MHz, $CDCl_3$) 175.3 (*C*-1), 173.4 (*C*-7), 165.2 (*C*(O)-biotin), 148.7 (*C*-4'), 129.4 (*C*-1'), 122.5 (*C*H-2'), 114.2 (*C*H-3'), 62.7(*C*H-biotin), 60.9 (*C*H-biotin), 56.4 (*C*H-biotin), 41.1 (*C*H_3-N), 40.8 (*C*H_2-biotin), 39.7 (*C*H_2-6), 37.2 (*C*H_2-2), 36.4 (*C*H_2-11), 29.5 – 28.8 (*C*H_2-3, *C*H_2-9, *C*H_2-10), 27.0 (*C*H_2-11), 26.3 (*C*H_2-4 or *C*H_2-5), 26.0 (*C*H_2-4 or *C*H_2-5); IR (neat, v_{max}/cm^{-1}) 3283, 2927, 1697, 1645, 1518, 1458, 1260; HRMS (ESI): calcd. for $C_{24}H_{38}N_5O_3S$ $[M+H]^+$ 476.2690, found 476.2688.

***N*-[4-(Dimethylamino)phenyl]acetamide (minimal-RTA) 4**[6]

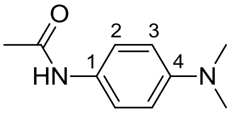

To a solution of *N,N*-dimethyl-*p*-phenylenediamine (100 mg, 0.73 mmol) in CH_2Cl_2 (0.5 mL) was added triethylamine (50 µL, 0.36 mmol) and *N,N*-dimethyl-4-aminopyridine (2 mg, 0.02 mmol), and the reaction mixture was stirred for 5 min at ambient temperature. Acetic anhydride (140 µL, 1.50 mmol) was added dropwise to the reaction mixture, and the resulting mixture was stirred for 2 hours at ambient temperature. The reaction was quenched with water (20 mL) and the product was extracted with CH_2Cl_2 (3 × 5 mL). The combined organic layers were washed with water (1 × 5 mL), dried over anhydrous Na_2SO_4, filtered and concentrated

in vacuo to give a brown solid. The crude product was purified by FCC (SiO_2, eluting with 50:50→100:0 EtOAc-Hex) to afford the *title compound* as a grey powder (89 mg, 68%). R_f 0.15 (EtOAc-Hexane 1:1); δ_H (400 MHz, DMSO-d$_6$) 9.58 (1H, s, N*H*), 7.42 – 7.31 (2H, app. d, *J* 9.1, 2-C*H*$_2$), 6.73 – 6.60 (2H, app. d, *J* 9.0, 3-C*H*$_2$), 2.83 (6H, s, N-C*H*$_3$), 1.97 (3H, s, C*H*$_3$); δ_C (100 MHz, DMSO-d$_6$) 167.3 (*C*=O), 146.9 (*C*-4), 129.3 (*C*-1), 120.5 (*C*H$_2$-2), 112.7 (*C*H$_2$-3), 40.5 (*C*H$_3$-N), 23.7 (*C*H$_3$); IR (neat, v_{max}/cm^{-1}) 3183, 2803, 1641, 1519, 1320; HRMS (ESI): calcd. for $C_{20}H_{28}N_4NaO_2$ $[2M+Na]^+$ 379.2106, found 379.2104. Data consistent with literature.

Expression and purification of proteins

Expression and purification of MCL-1, BCL-x$_L$, *h*DM2 and MCL-1 Cys286Ser protein was performed as previously described.[5,7,8,9]

Sodium dodecyl sulfate-polyacrylamide gel electrophoresis (SDS-PAGE)

A resolving gel of appropriate percentage was prepared according to Supplementary Table 4: 12% gels were used in analysis of MCL-1 expression and purification, 15% gels were used in the analysis of all photolabelling reactions. Following addition of tetramethylethylenediamine (TEMED), the solution was thoroughly mixed and immediately added to the BioRad tetragel apparatus. A layer of propan-2-ol (1 mL) was applied to the top of the gel. When the resolving gel had set, the propan-2-ol was removed and a stacking gel was prepared according to Supplementary Table 4. Once the TEMED was added the solution was thoroughly mixed, immediately added to the top of the resolving gel and comb with a suitable number of lanes was inserted into the stacking gel.

Supplementary Table 4: *SDS-PAGE gel recipes*

**Components**	**Resolving gel**		**Stacking gel**
	**12%**	**15%**	
1.5 M Tris (pH 8.8)	2.53 mL	2.53 mL	-
0.5 M Tris (pH 6.8)	-	-	0.95 mL
40 % (w/v) acrylamide	3.00 mL	3.75 mL	0.62 mL
10% SDS	100 µL	100 µL	50 µL
H_2O	4.25 mL	3.50 mL	3.30 mL
10% APS	100 µL	100 µL	50 µL
TEMED	10 µL	10 µL	10 µL

Once set, the gel was placed inside an electrophoresis tank and SDS running buffer was added. Samples were mixed with an equal volume of loading buffer, heated to 95 °C for 10 minutes and loaded onto the gel. Electrophoresis was performed at a constant voltage of 180V for approximately 50 minutes, or until the loading buffer had reached the bottom of the gel. Proteins were visualised using Coomassie Blue stain (45% (v/v) methanol, 10% (v/v) acetic acid, 0.25% (w/v) Coomassie Brilliant Blue R-250) and destained (30% (v/v) methanol, 10% (v/v) acetic acid), or alternatively using Instant Blue (TripleRed).

Fluorescence anisotropy assays – general remarks

Fluorescence anisotropy assays were run in 384 well Optiplates and scanned using a Perkin Elmer EnVision™ 2103 MultiLabel plate reader. Fluorescein labelled peptides used an excitation and emission wavelength of 480 nm (30 nm bandwidth) and 535 nm (40 nm bandwidth) respectively. All assays were run in Tris buffer (50 mM Tris, 150 mM NaCl, 0.01% Triton X-100, pH 7.4) with additives where described.

Direct fluorescence anisotropy binding assays – data processing

The data obtained for both the *P* (perpendicular intensity) and *S* (parallel intensity) channels were corrected by subtracting the corresponding control wells, and the resulting values were used to calculate intensity (Eq. 1) and anisotropy (Eq. 2) for each well (using Microsoft Excel). These data were transferred into OriginPro 8.5, where a plot of anisotropy against protein concentration was fitted using a logistic model (Eq. 3) to obtain the minimum (r_{min}) and maximum (r_{max}) values of anisotropy. These values were used to determine the fraction of labelled peptide bound to the protein (fraction ligand bound, L_b, Eq. 4), and fitted (Eq. 5) in OriginPro 8.5 to determine the dissociation constant, K_d.

$$I = 2PG + S \qquad \textit{Equation 1}$$

Where *I* is the total intensity, *G* is an instrument factor which was set to 1, and *r* is the anisotropy.

$$r = \frac{S - PG}{I} \qquad \textit{Equation 2}$$

$$y = r_{max} + \frac{r_{min} - r_{max}}{1 + \left({}^{x}/_{x_0}\right)^{p}} \qquad \textit{Equation 3}$$

$$Lb = \frac{r - r_{min}}{(\lambda(r_{max} - r) + r - r_{min})} \qquad \textit{Equation 4}$$

Where x_0 is the midpoint, *p* is the power and λ is the ratio of $I_{bound}/I_{unbound}$ and is equal to 1.

$$y = \frac{(K_d + x + [FL]) - \sqrt{((K_d + x + [FL])^2 - 4x[FL])}}{2} \qquad \textit{Equation 5}$$

Where *Lb* is the fraction ligand bound, [FL] is the concentration of fluorescent ligand, y is Lb*[FL], *x* = [added titrant].

MCL-1/FITC-NOXA-B direct titration

Titration of MCL-1 into NOXA-B was performed in a 384 well plate in Tris Buffer (50 mM Tris, 150 mM NaCl, 0.01% Triton X-100, pH 7.4) with the concentration of MCL-1 starting at 10 µM, diluted over 24 points in a 1/2 regime with [FITC-NOXA-B] fixed at 25 nM. Plates were read after 1 hour and 20 hours incubation. Assays were run in triplicate (both test wells and control wells).

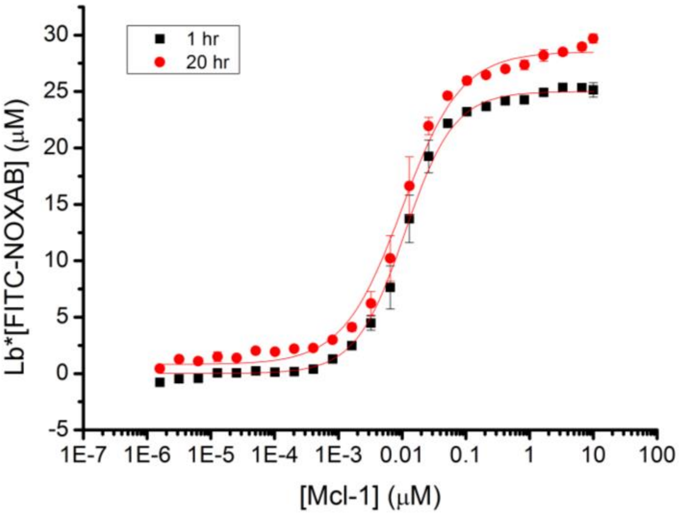

Supplementary Figure 11: *Direct titration of MCL-1 into FITC-NOXA-B to give a K_d of 11 ± 3 nM (error stated is SEM) that corresponds to that described previously (13 ± 2 nM).*[9] *Error bars represent the standard deviation of three replicates.*

BCL-X_L/FITC-BID direct titration

Titration of BCL-X_L into FITC-BID was performed in a 384 well plate in Tris Buffer (50 mM, Tris, 150 mM NaCl, 0.01% Triton-X-100, pH 7.4) + 0.02 mg/mL BSA with the concentration of BCL-X_L starting from 10 µM, diluted over 24 points in a 1/2 regime with [FITC-BID] fixed at 25 nM. Plates were read after 1 hour incubation and 20 hours incubation, data shown is from 1 hour incubation (assay died at 20 hour time point). Assays were run in triplicate (both test wells and control wells).

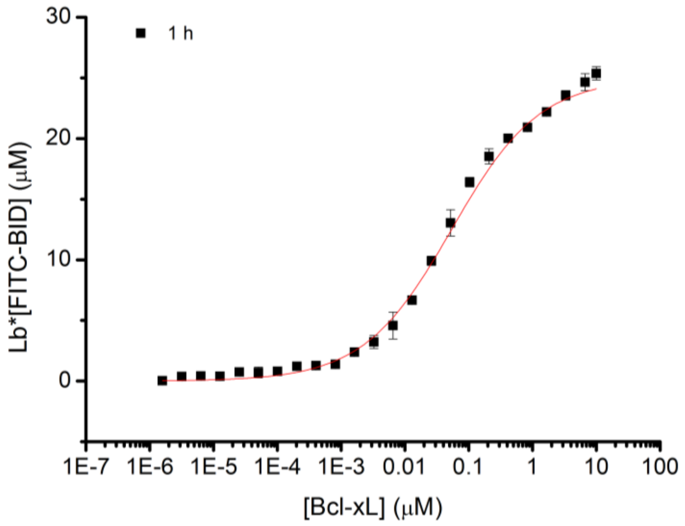

Supplementary Figure 12: *Direct titration of BCL-X_L into a constant concentration of FITC-BID to give a K_d of 54 ± 8 nM (error stated is SEM) that corresponds to that described previously (79 ± 6 nM).*[5] *Error bars represent the standard deviation of three replicates.*

Fluorescence anisotropy competition assays - general remarks

All competition assays were performed in 384 well plates with the concentration of peptide competitor serially diluted over 16 or 24 points in a 1/2 regime with [tracer] fixed at 25 nM. The assays consisted of three test rows (containing protein, tracer and competitor), and three control rows (tracer peptide was replaced with buffer). The intensity, *I*, and anisotropy, *r*, were calculated using equations 1 and 2, respectively. A plot of anisotropy against competitor concentration was plotted in OriginPro 8.5 and fitted using Eq. 4 to determine an IC_{50} value.

Competition of MCL-1/FITC-NOXA-B interaction by Ru(II)(bpy)$_3$-NOXA-B 1 and Ac-NOXA-B 5

MCL-1/FITC-NOXA-B competition assays were performed in Tris Buffer (50 mM, Tris, 150 mM NaCl, pH 7.4) + 0.01% Triton-X-100 with [MCL-1] fixed at 75 nM. Assays were run in triplicate (both test wells and control wells). Plates were read after 1 h and 20 h incubation.

Competition of BCL-X$_L$/FITC-BID 7 interaction by Ru(II)(bpy)$_3$-NOXA-B 1

BCL-X$_L$/FITC-BID competition assays were performed in Tris Buffer (50 mM, Tris, 150 mM NaCl, 0.01% Triton-X-100, pH 7.4) + 0.02 mg/mL BSA with [BCL-X$_L$] fixed at 50 nM. Both control wells and test wells were run in triplicate. Plates were read after 1 h and 20 h incubation, data shown is from 20 h incubation.

Competition of MCL-1/FITC-NOXA-B interaction by Ac-BID variant 6b and Ac-BID

MCL-1/FITC-NOXA-B competition assays were performed in Tris Buffer (50 mM, Tris, 150 mM NaCl, pH 7.4) + 0.01% Triton-X-100 with [MCL-1] fixed at 75 nM. Assays were run in triplicate (both test wells and control wells). Plates were read after 1 h and 20 h incubation.

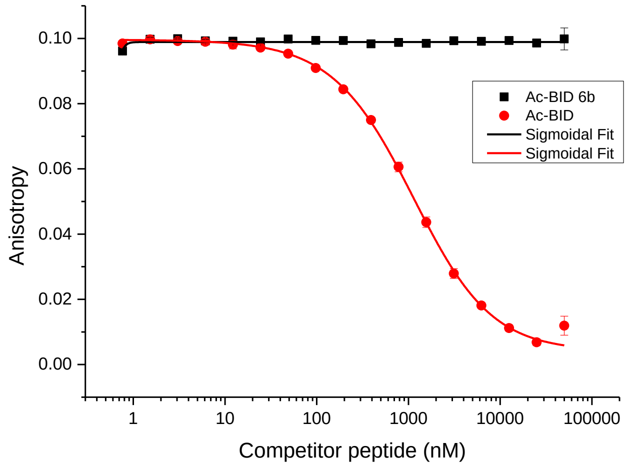

Supplementary Figure 13: *Fluorescence anisotropy competition titration for the inhibition of the MCL-1/FITC-NOXA-B interaction by Ac-BID variant **6b** (black) and Ac-BID **6a** (red). Error bars represent the standard deviation of three repeats.*

Ligand-directed photolabelling of recombinant MCL-1 with TAMRA-RTA 2

To a solution of MCL-1 (final concentration 5 μM) in ammonium acetate buffer (50 mM, pH 7.5) was added Ru(II)(bpy)$_3$-NOXA-B **1** (final concentration 1 μM), TAMRA-RTA **2** (final concentration 5-10 μM, unless stated otherwise) and APS (final concentration 10 μM), and the mixture was incubated at r.t. for 5 min. For the competition experiments, Ac-BID **6a** or Ac-BID variant **6b** (final concentration 0-1000 μM; DMSO concentrations were adjusted in all wells) was added and the mixture was incubated for 10 min prior to incubation with Ru(II)(bpy)$_3$-NOXA-B. The mixture was irradiated for 1 min at r.t., 5 cm from the light source (Kessil H150W-BLUE LED, 32W, 2 × lamps). The reaction was immediately quenched by the addition of DTT (final concentration 10 mM) and analysed using ESI-MS and/or SDS-PAGE. Fluorescently modified peptides were analysed using a Molecular Imager ChemiDoc XRS System (Bio-Rad, CA). After obtaining the fluorescence image, the same gel was stained with Coomassie Brilliant Blue (CBB) and visualised on a Molecular Imager ChemiDoc XRS System. ImageJ was used to quantify bands on the gel.

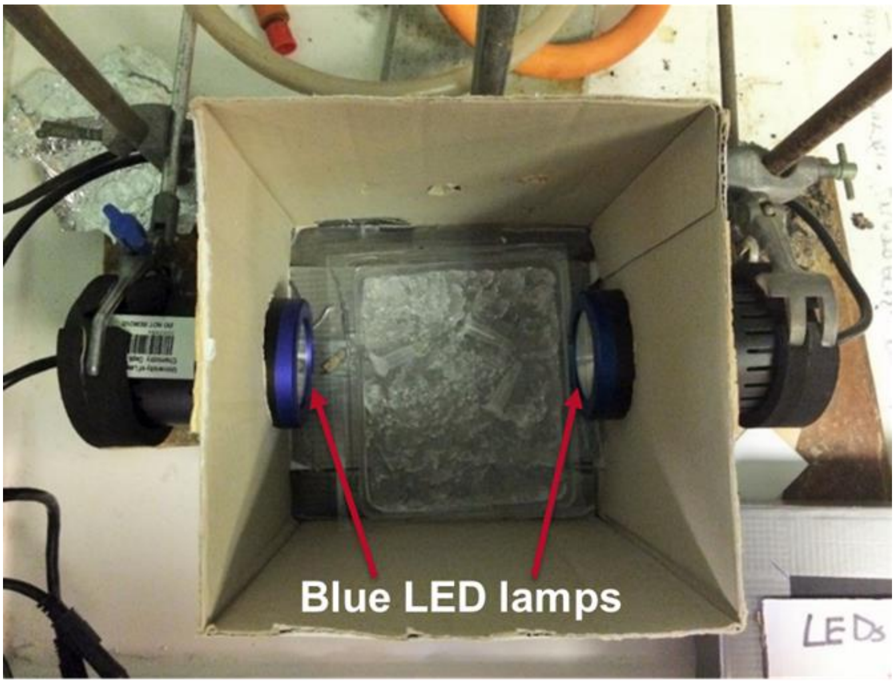

Supplementary Figure 14: *Photolabelling set-up: two Kessil H150W-BLUE LED lamps facing each other, parallel to base of the box containing the samples in Eppendorf tubes.*

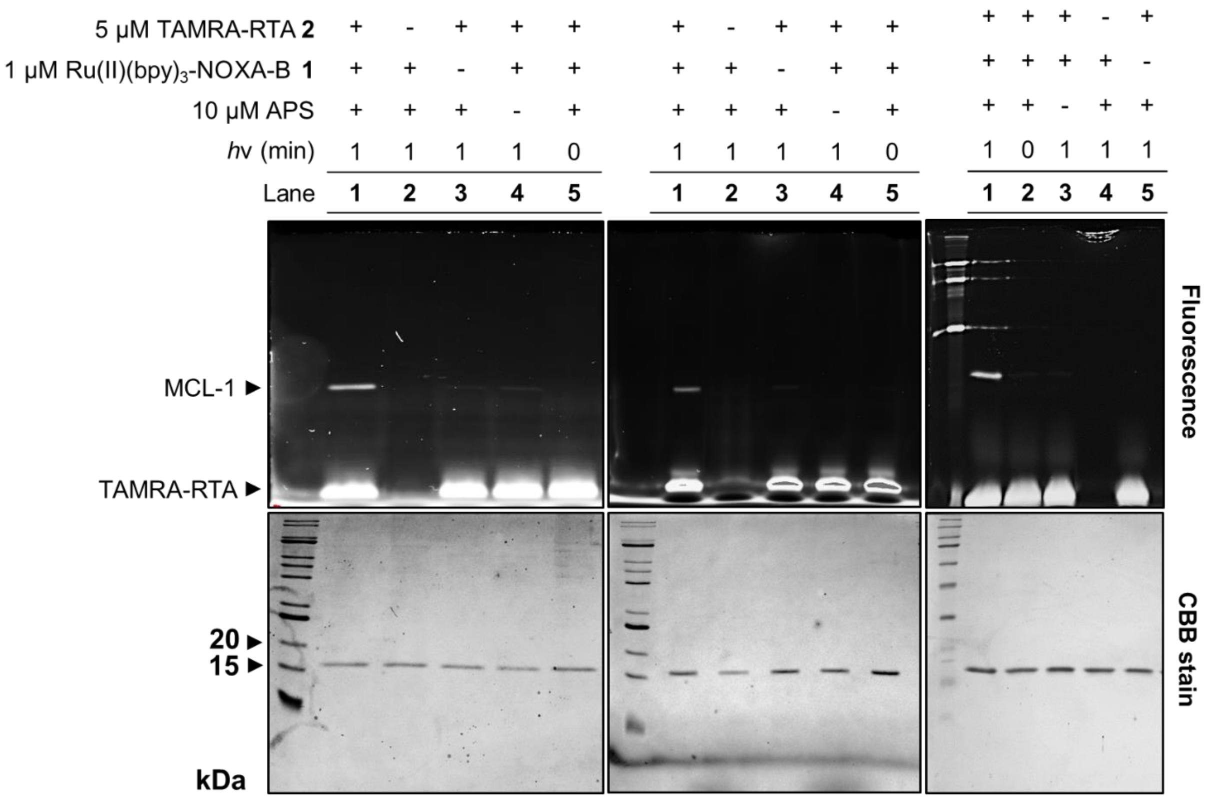

Supplementary Figure 15: *Full gels for photolabelling experiments of MCL-1 with TAMRA-RTA **2** displayed in Fig. 2c (3 independent repeats) Fluorescence image (top) and Coomassie Brilliant Blue (CBB, bottom) stained SDS-PAGE gels; fluorescence image shows MCL-1 only labelled when 1 min hv, 5 µM TAMRA-RTA and 1 µM Ru(II)(bpy)$_3$-NOXA-B are present (5 µM MCL-1 and 10 µM APS used), CBB stain shows MCL-1 for all conditions.*

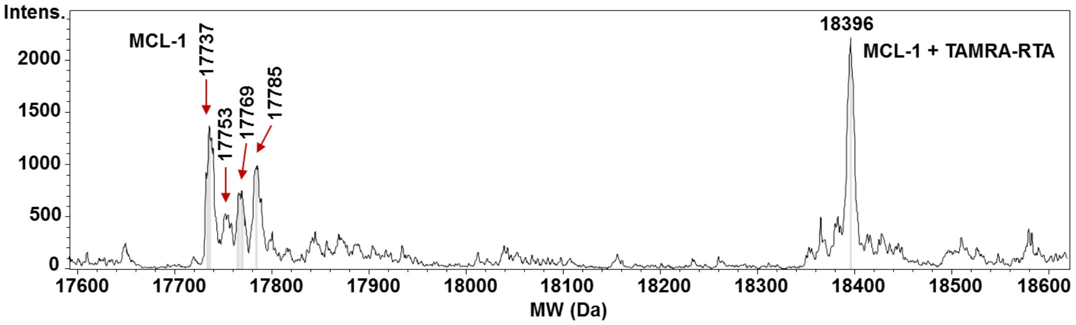

Supplementary Figure 16: *ESI-MS data showing photolabelling of MCL-1 with TAMRA-RTA **2** after 1 minute of irradiation. Conditions: 5 µM protein, 1 µM Ru(II)(bpy)$_3$-NOXA-B, 5 µM TAMRA-RTA, 10 µM APS, 50 mM (NH$_4$)HCO$_3$ (pH 7.4). Masses indicative of oxidation of amino acid residues on unlabelled MCL-1 are present in the mass spectrum (mass increments of +16 Da).*

Labelling of recombinant MCL-1 with Ac-RTA 4

To a solution of MCL-1 (5 μM) in ammonium acetate buffer (50 mM, pH 7.5) was added Ru(II)(bpy)$_3$-NOXA-B (final concentration 1 μM), Ac-RTA **4** (final concentration 50 μM) and APS (final concentration 10 μM) and the mixture was incubated at r.t. for 5 min. The mixture was irradiated for 1 min at r.t., 5 cm from light source (Kessil H150W-BLUE LED, 32W, 2 × lamps). The reaction was immediately quenched by the addition of DTT (final concentration 10 mM) and analysed using ESI-MS and/or SDS-PAGE. For samples analysed using HR-ESI-MS, labelling of MCL-1 with Ac-RTA **4** was quantified by measuring the absolute ion counts of the unlabelled MCL-1 (17736 Da) and labelled MCL-1 (17912 Da) in the deconvoluted mass spectra, processed using Bruker compass data analysis software.

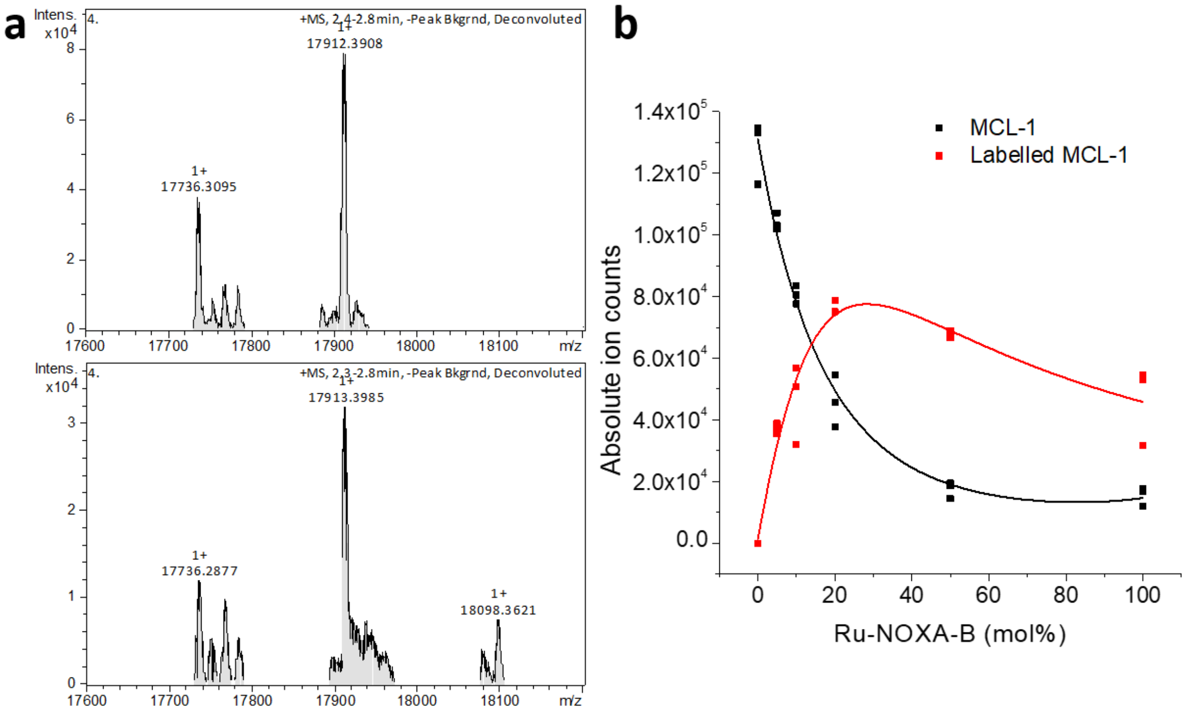

Supplementary Figure 17: *Photolabelling of MCL-1 with Ac-RTA **4** mediated by Ru(II)(bpy)$_3$-NOXA-B **1**. (a) Deconvoluted ESI-MS spectra showing photolabelling of MCL-1 with Ac-RTA **4** using 20 mol% (top) and 100 mol% (bottom) Ru(II)(bpy)$_3$-NOXA-B **1**. MCL-1 (17736 Da) and photolabelled MCL-1 (17912 Da). (b) Graph showing the absolute ion count of MCL-1 and photolabelled MCL-1 detected by ESI-MS following analysis of photolabelling reaction mixtures at increasing concentrations of Ru(II)(bpy)$_3$-NOXA-B **1** (n = 3 independent experiments per concentration of **1**). Conditions: 5 μM MCL-1, 0-5 μM Ru(II)(bpy)$_3$-NOXA-B **1**, 50 μM Ac-RTA **4**, 10 μM APS, 1 min irradiation, 50 mM (NH$_4$)HCO$_3$ (pH 7.4).*

Labelling of recombinant MCL-1 with biotin-RTA 3

To a solution of MCL-1 (5 μM) in ammonium acetate buffer (50 mM, pH 7.5) was added Ru(II)(bpy)$_3$-NOXA-B (final concentration 1 μM), biotin-RTA (final concentration 5 μM) and APS (final concentration 10 μM) and the mixture was incubated at r.t. for 5 min. The mixture was irradiated for 1 min at r.t., 5 cm from light source (Kessil H150W-BLUE LED, 32W, 2 × lamps). The reaction was immediately quenched by the addition of DTT (final concentration 10 mM) and analysed using ESI-MS and/or SDS-PAGE.

ESI-MS spectra of MCL-1 labelling reactions using biotin-RTA

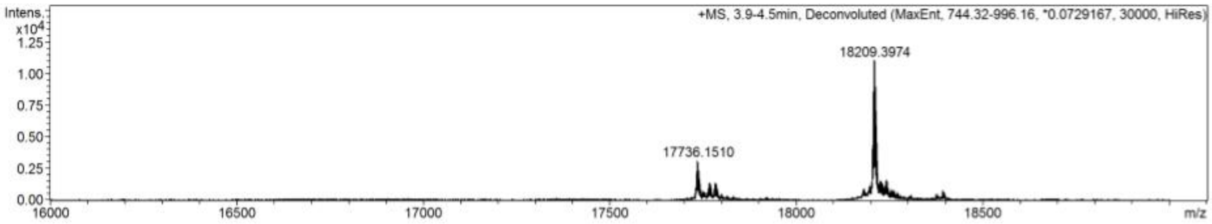

Conditions: 5 µM MCL-1, 1 µM Ru(II)(bpy)$_3$-NOXA-B **1**, 5 µM biotin-RTA **3**, 10 µM APS, 1 min *h*ν.

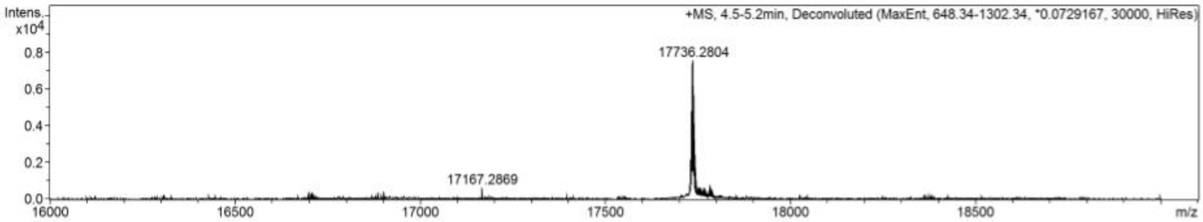

Conditions: 5 µM MCL-1, 0 µM Ru(II)(bpy)$_3$-NOXA-B **1**, 5 µM biotin-RTA **3**, 10 µM APS, 1 min *h*ν.

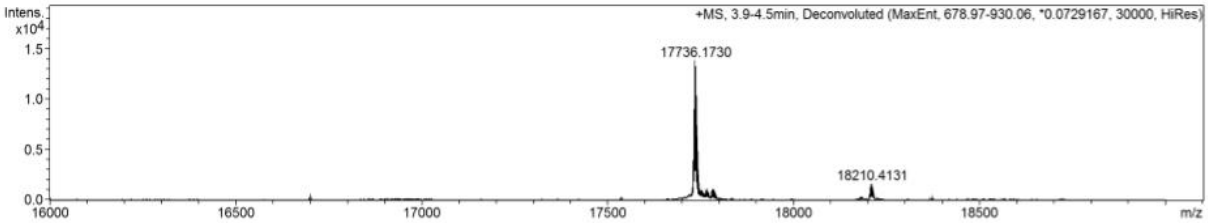

Conditions: 5 µM MCL-1, 1 µM Ru(II)(bpy)$_3$-NOXA-B **1**, 5 µM biotin-RTA **3**, **0** µM APS, 1 min *h*ν.

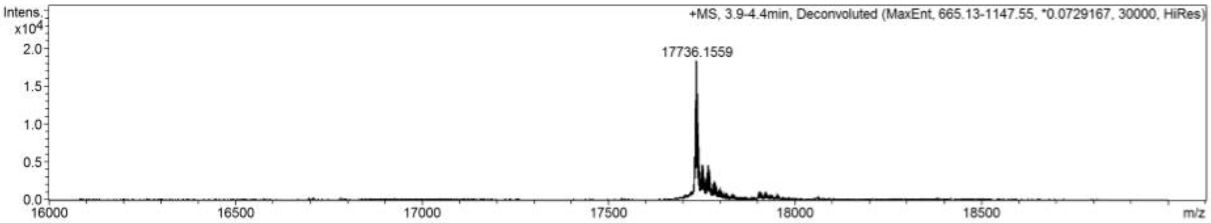

Conditions: 5 µM MCL-1, 1 µM Ru(II)(bpy)$_3$-NOXA-B **1**, **0** µM biotin-RTA **3**, 10 µM APS, 1 min *h*ν.

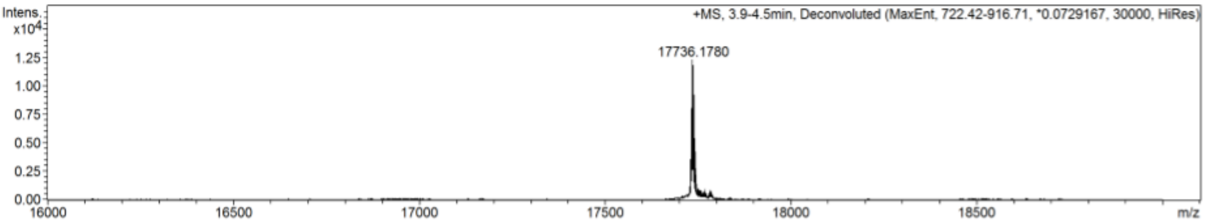

Conditions: 5 µM MCL-1, 1 µM Ru(II)(bpy)$_3$-NOXA-B **1**, 5 µM biotin-RTA **3**, 10 µM APS, 0 min *h*ν.

Supplementary Figure 18: *Deconvoluted ESI-MS spectra from photolabelling reactions under various conditions. Notably, the reaction without Ru(II)(bpy)$_3$-NOXA-B **1** gives no biotin-labelled protein.*

Pull-down of biotinylated MCL-1

The crude labelling mixture was incubated with avadin-agarose beads (50 µL) at r.t. for 2 hours with gentle agitation (input lanes 1-4 and their respective pull-down lanes 6-9). Note that for input lane 5/pull-down lane 10, excess biotin-RTA **3** was removed from the crude labelling mixture using a protein concentrator (10 kDa MWCO) prior to incubation with avidin-agarose beads. Following removal of the supernatant, the beads were washed twice with PBS (100 µL), then boiled at 90 °C for 10 mins in sample loading buffer to elute bound proteins. Samples were separated by SDS-PAGE: the gel (15% acrylamide) was run at a constant voltage of 180 V for 45 min, then the gel was stained with Coomassie Brilliant Blue (CBB) and the image obtained on a Molecular Imager ChemiDoc XRS.

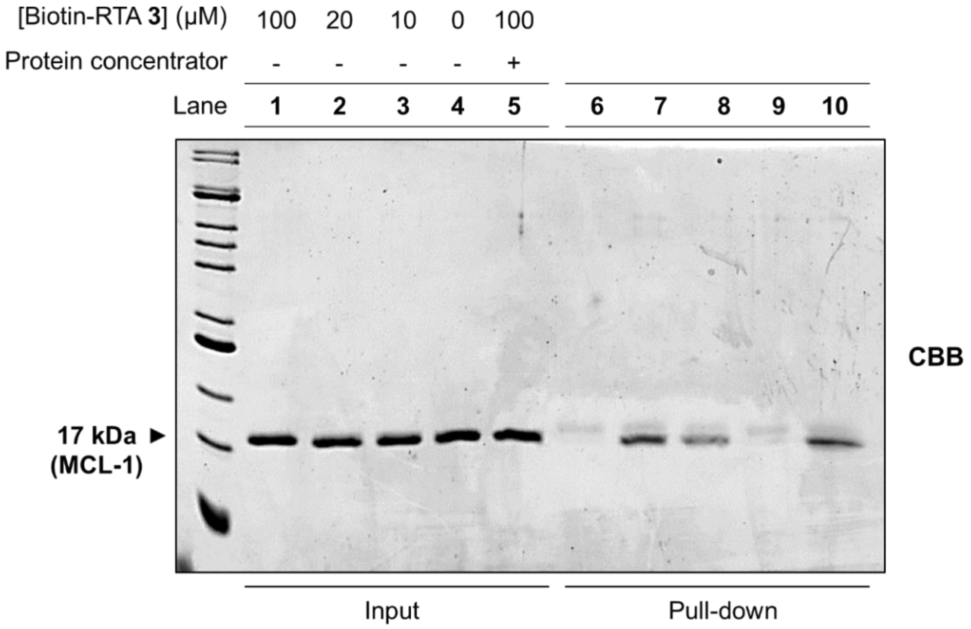

Supplementary Figure 19: *Isolation of biotinylated MCL-1 from crude labelling mixture through pull-down with avadin-agarose beads (CBB stained gel). Conditions: 5 µM MCL-1, 0-100 µM biotin-RTA* ***3****, 1 µM Ru(II)(bpy)*$_3$*-NOXA-B, 10 µM APS, 1 min irradiation, 50 mM (NH*$_4$*)HCO*$_3$ *(pH 7.4). Biotinylated MCL-1 was pulled-down when 20 µM and 10 µM biotin-RTA* ***3*** *was used (lanes 7 and 8, respectively), in addition to when 100 µM biotin-RTA* ***3*** *was used and the excess label* ***3*** *was removed using a protein concentrator (lane 10). 50 µL avidin-agarose beads was used in pull-down experiments.*

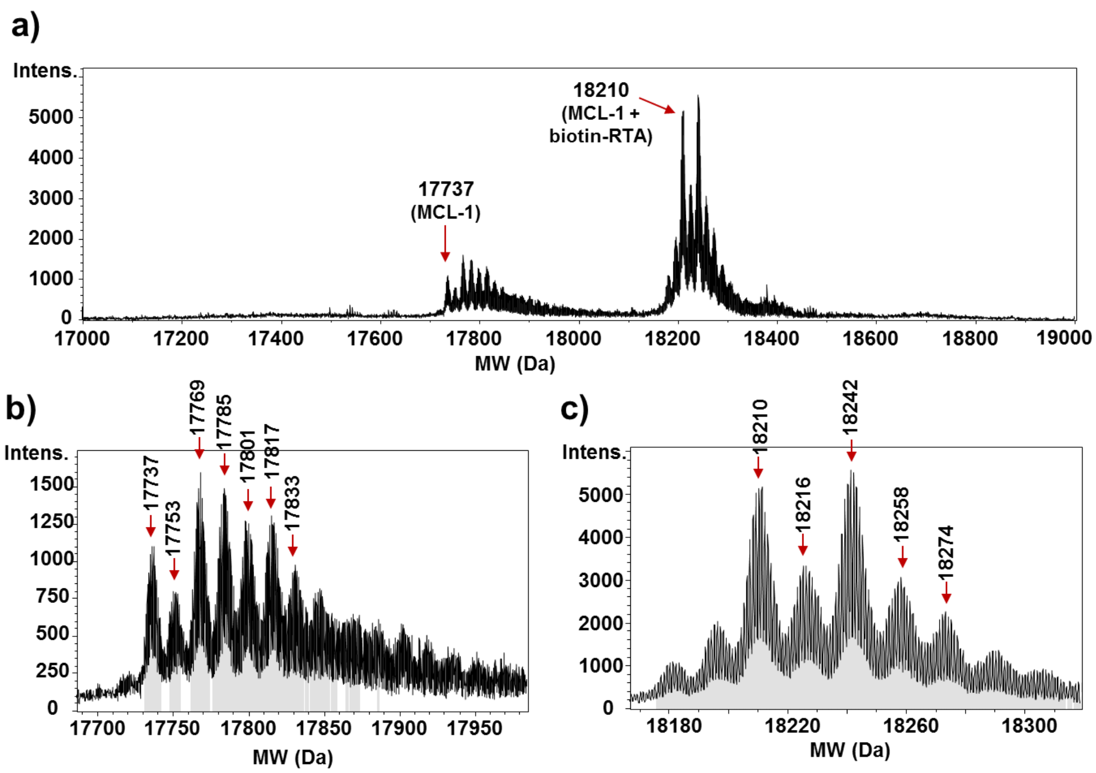

Supplementary Figure 20: *a) ESI-MS data showing photolabelling of MCL-1 with biotin-RTA **3** after 15 mins irradiation. Conditions: 5 µM protein, 1 µM Ru(II)(bpy)$_3$-NOXA-B, 5 µM biotin-RTA **3**, 10 µM APS, 50 mM $(NH_4)HCO_3$ (pH 7.4). b) Zoom in on MCL-1 peak, showing mass increments of +16 Da, indicating oxidation of amino acid residues. b) Zoom in on labelled MCL-1 peak, showing masses +16 Da, indicating oxidation of amino acid residues.*

Determination of labelling site by peptide mapping of MCL-1

To a solution of MCL-1 (50 µM) in ammonium acetate buffer (50 mM, pH 7.5) was added Ru(II)(bpy)$_3$-NOXA-B **1** (final concentration 10 µM), Ac-RTA **4** (final concentration 10 µM) and APS (final concentration 10 µM) and the mixture was incubated at r.t. for 5 min. The mixture was irradiated for 1 min at r.t., 5 cm from light source (Kessil H150W-BLUE LED, 32W, 2 × lamps) and immediately quenched by the addition of DTT (final concentration 10 mM). The sample was split into two 50 µL aliquots. To each aliquot, a protease solution (Trypsin or Glu-C; Promega (Madison, WI); 20 ng µL^{-1} in 25 mM ammonium bicarbonate) was added in a 1:50 ratio (protease:total protein content). Samples were incubated at 37 ºC with shaking for 18 h. The digest reaction was stopped by adding 5 µL of 1% HCOOH, then subjected to purification using a Sep-pak 18 column. The Sep-pak column was equilibrated with 1 mL 0.1% TFA. 500 µL of 0.1% TFA was added to the peptide digest, the mixture was passed through the column and the column was washed with 1 mL 0.1% TFA. Peptides were eluted from the column with 500 µL MeCN-H_2O 1:1 + 0.1% HCOOH. The eluant was dried by vacuum centrifugation and the peptides were reconstituted in 20 µL 0.1% TFA. LC separation of the peptide mixtures was performed on an ACQUITY M-Class UPLC (Waters UK, Manchester). 1 µL sample was loaded onto a Symmetry C18 trap column and washed with 1% MeCN/0.1% HCOOH for 5 min at 5 µL min^{-1}, then the peptides were separated on a HSS T3 C18 analytical column (Waters UK, Manchester) by gradient elution of 1-60% solvent B (0.1% HCOOH in MeCN) in A (0.1% HCOOH in H_2O) over 30 min at 0.3 µL min^{-1}. The column eluant was directly interfaced to a quadrupole-orthogonal time of flight mass spectrometer (Xevo G2-XS Q-TOF, Waters UK, Manchester) via a Z-spray nanoflow electrospray source. The MS was operated in positive TOF mode using a capillary voltage of 3.0 kV, cone voltage of 40 V, source offset of 80 V, backing pressure of 3.58 mbar. The source temperature was 80 °C. Argon was used as the buffer gas at a pressure of 8.6 × 10^{-3} mbar in the trap and transfer regions. Mass calibration was performed using [Glu]-fibrinopeptide (GFP) at a concentration of 250 fmol µL^{-1}. GFP was also used as a lock mass calibrant with a one second lock spray scan taken every 30 s during acquisition. Ten scans were averaged to determine the lock mass correction factor. Data acquisition was using data dependent analysis with a 0.2 s scan MS over *m/z* 350-2000 being followed by five 0.5 s MS/MS taken of the five most intense ions in the MS spectrum. CE applied was dependent upon charge state and mass of the ion selected. Dynamic exclusion of 60 s was used. Data processing was performed using the MassLynx v4.1 suite of software supplied with the mass spectrometer. Peptide MS/MS data were processed with PEAKS Studio (Bioinformatic Solutions Inc, Waterloo, Ontario, Canada) and searched against the amino acid sequence. 176.0951 Da was set as a variable modification on any residues to

determine the position of the Ac-RTA **4** modification. MS mass tolerance was 10 ppm, and fragment ion mass tolerance was 0.05 Da. The false discovery rate was set to 1%.

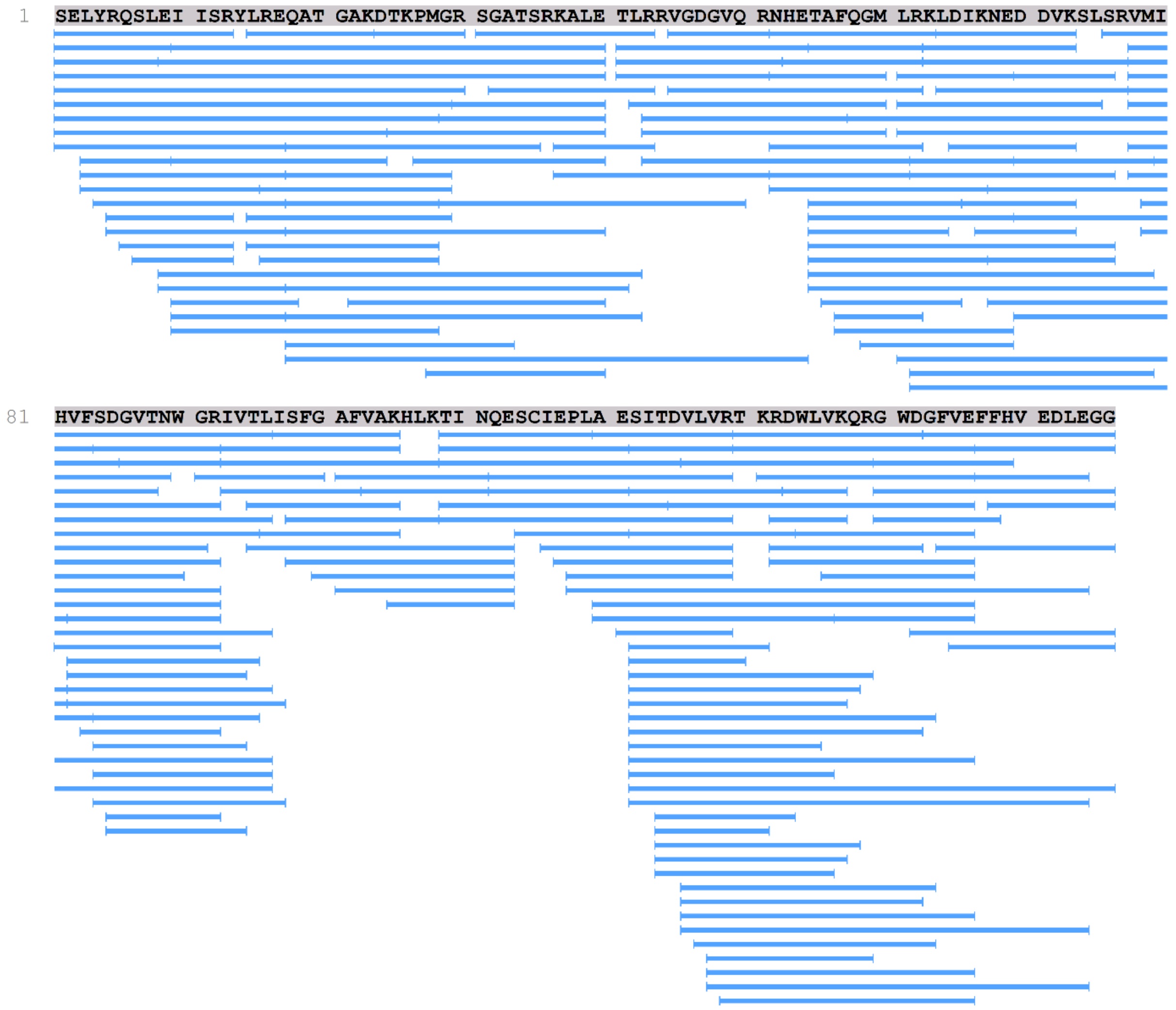

Supplementary Figure 21: *Peptide fragments (shown in blue) detected by LC-MS/MS upon digestion of unmodified MCL-1 using Glu-C and trypsin proteases. Sequence coverage 97%.*

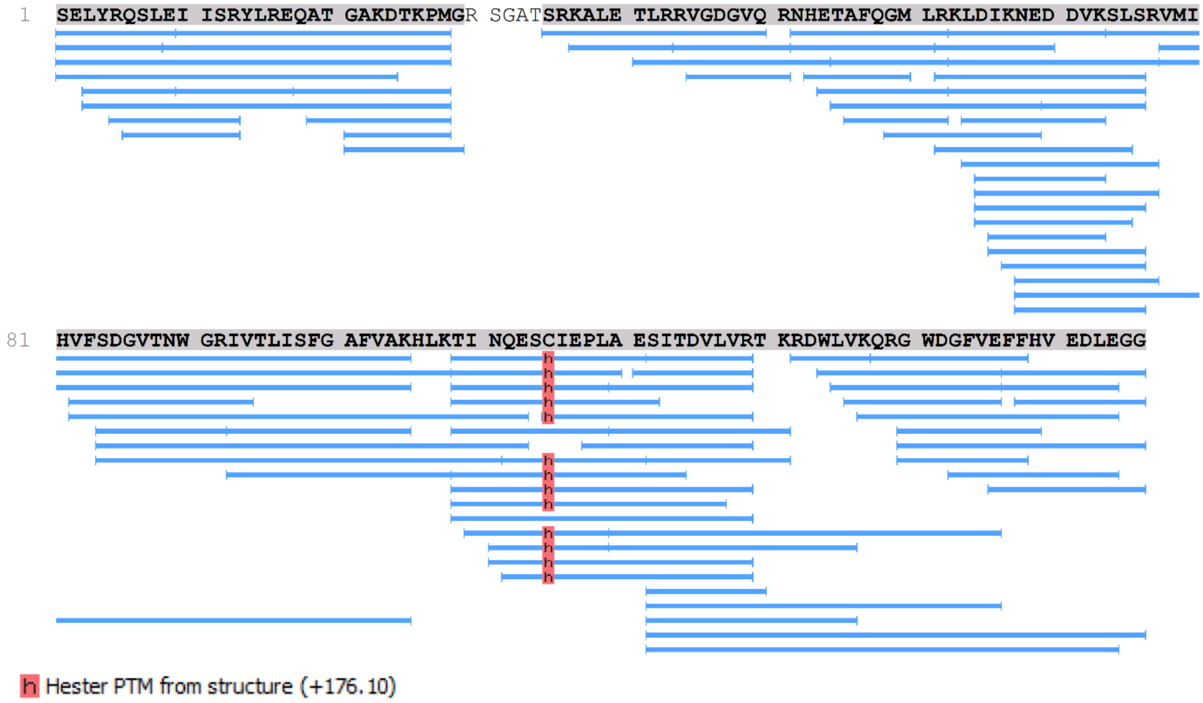

Supplementary Figure 22: *Peptide fragments (shown in blue) detected by LC-MS/MS upon digestion of MCL-1 modified with Ac-RTA **4**, using Glu-C and trypsin proteases. Sequence coverage 94%. 'h' denotes the position of the additional mass of 176.10 Da on the modified peptides.*

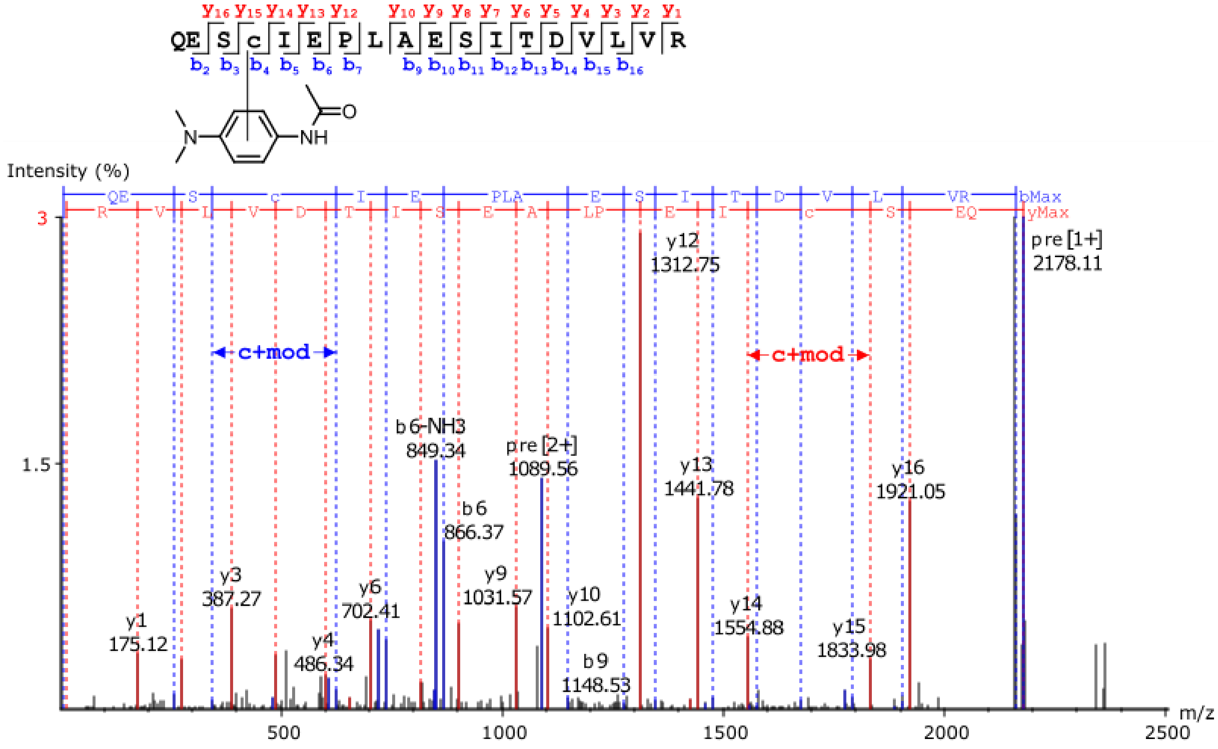

Supplementary Figure 23: *Q-TOF MS/MS spectrum for a selected peptide modified with Ac-RTA **4**. Observed y and b ions are shown in red and blue, respectively. Modification is found on residue Cys286.*

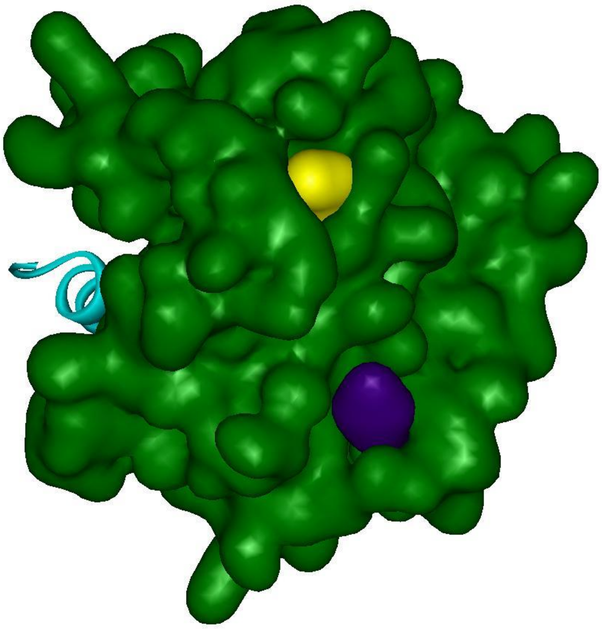

Supplementary Figure 24: *Space-filling representation of MCL-1/NOXA-B structure (PDB:2NLA), highlighting Cys286 (yellow) and Tyr175 (purple). Tyr185 cannot be seen, suggesting it is not accessible. MCL-1 is shown in green; NOXA-B peptide is shown in cyan.*

Photolabelling experiments with MCL-1 Cys286Ser

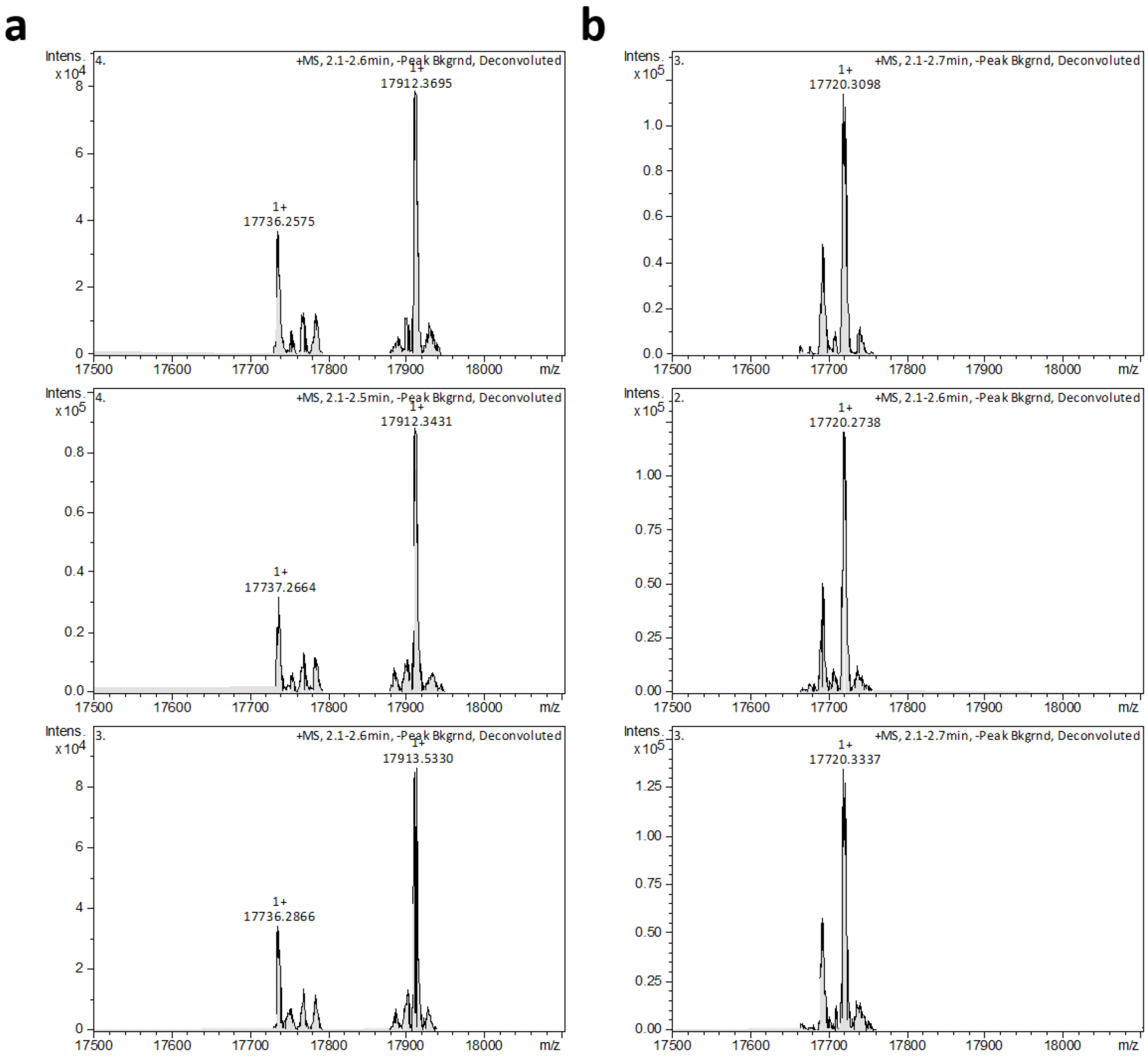

Supplementary Figure 25: *Photolabelling of MCL-1 and MCL-1 Cys286Ser with Ac-RTA **4** mediated by Ru(II)(bpy)$_3$-NOXA-B **1** (3 independent repeats each). (a) Deconvoluted ESI-MS spectra for MCL-1 photolabelling reaction mixtures displaying MCL-1 (17736 Da) and photolabelled MCL-1 (17912 Da). Conditions: 5 µM MCL-1, 1 µM Ru(II)(bpy)$_3$-NOXA-B **1**, 50 µM Ac-RTA **6**, 10 µM APS, 1 min irradiation, 50 mM (NH$_4$)HCO$_3$ (pH 7.4). (b) Deconvoluted ESI-MS spectra for MCL-1 Cys286Ser variant photolabelling reaction mixtures displaying MCL-1 Cys286Ser variant (17720 Da). Conditions: 5 µM MCL-1 Cys286Ser, 1 µM Ru(II)(bpy)$_3$-NOXA-B **1**, 50 µM Ac-RTA **4**, 10 µM APS, 1 min irradiation, 50 mM (NH$_4$)HCO$_3$ (pH 7.4).*

Exploring the ligand-directed nature of MCL-1 labelling with non-targeted SET reagent Ru(II)(bpy)$_3$

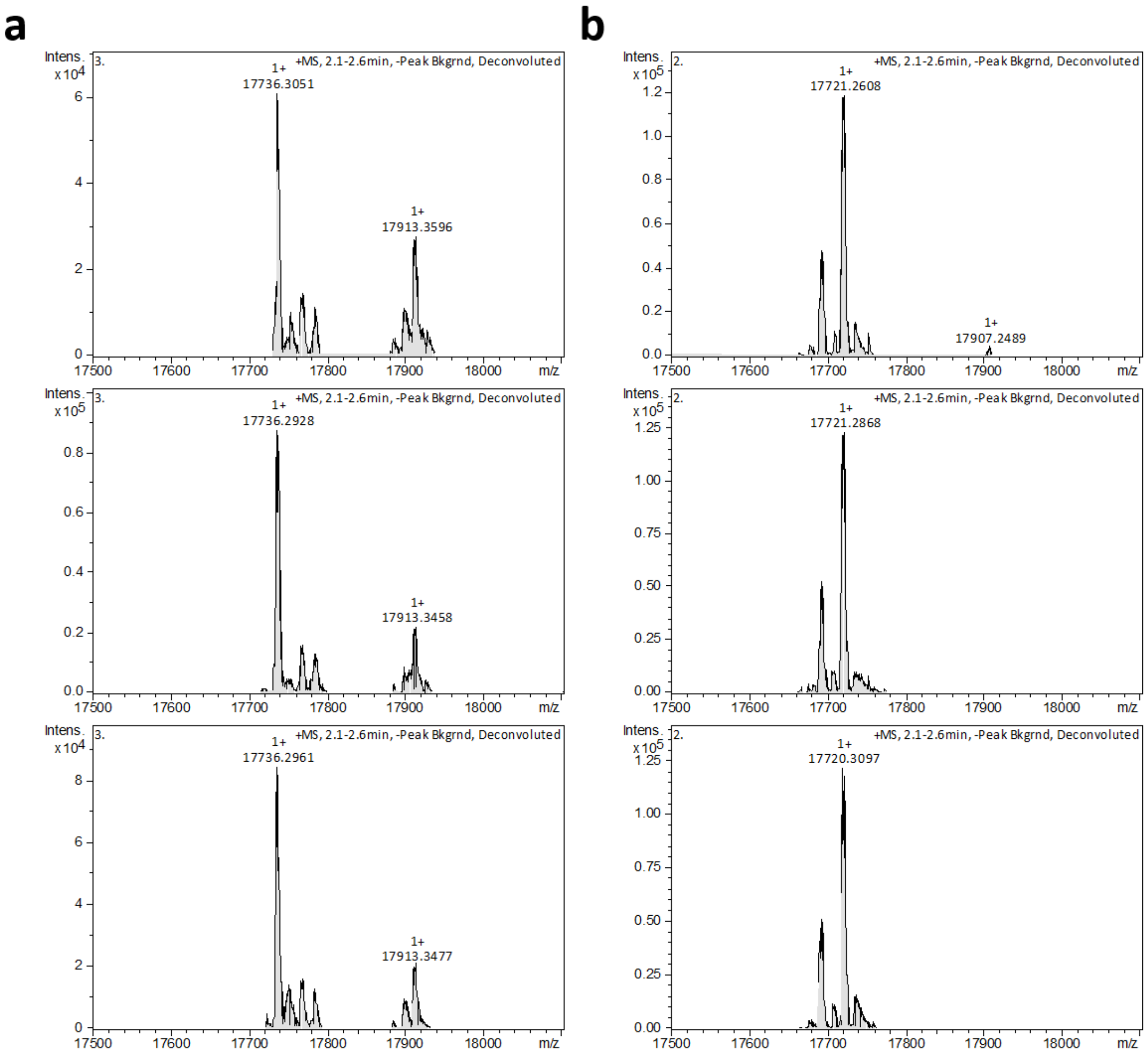

Supplementary Figure 26: *Photolabelling of MCL-1 and MCL-1 Cys286Ser with Ac-RTA **4** mediated by Ru(II)(bpy)$_3$. (a) Deconvoluted ESI-MS spectra for MCL-1 photolabelling reaction mixtures displaying MCL-1 (17736 Da) and photolabelled MCL-1 (17912 Da). Conditions: 5 μM MCL-1, 1 μM Ru(II)(bpy)$_3$, 50 μM Ac-RTA **4**, 10 μM APS, 1 min irradiation, 50 mM (NH$_4$)HCO$_3$ (pH 7.4). (b) Deconvoluted ESI-MS spectra for MCL-1 Cys286Ser photolabelling reaction mixtures displaying MCL-1 Cys286Ser variant (17720 Da). Conditions: 5 μM MCL-1 Cys286Ser variant, 1 μM Ru(II)(bpy)$_3$, 50 μM Ac-RTA **4**, 10 μM APS, 1 min irradiation, 50 mM (NH$_4$)HCO$_3$ (pH 7.4).*

Competition experiments using Ac-NOXA-B and Ac-BID with <u>targeted SET reagent</u> Ru(II)(bpy)3-NOXA-B 1

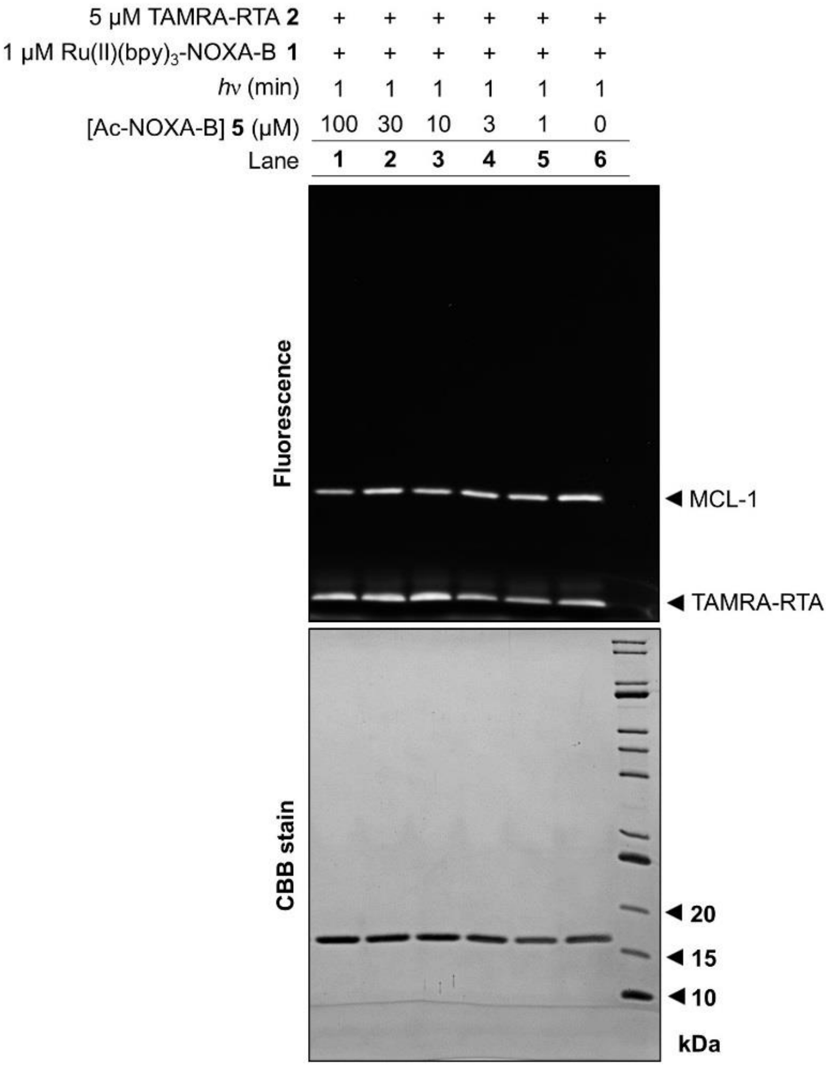

Supplementary Figure 27: *Competition experiment using Ac-NOXA-B **5**. Conditions: 5 µM MCL-1, 5 µM TAMRA-RTA **2**, 1 µM Ru(II)(bpy)$_3$-NOXA-B **1**, 10 µM APS, 1 min hv, 0-100 µM Ac-NOXA-B **5**.*

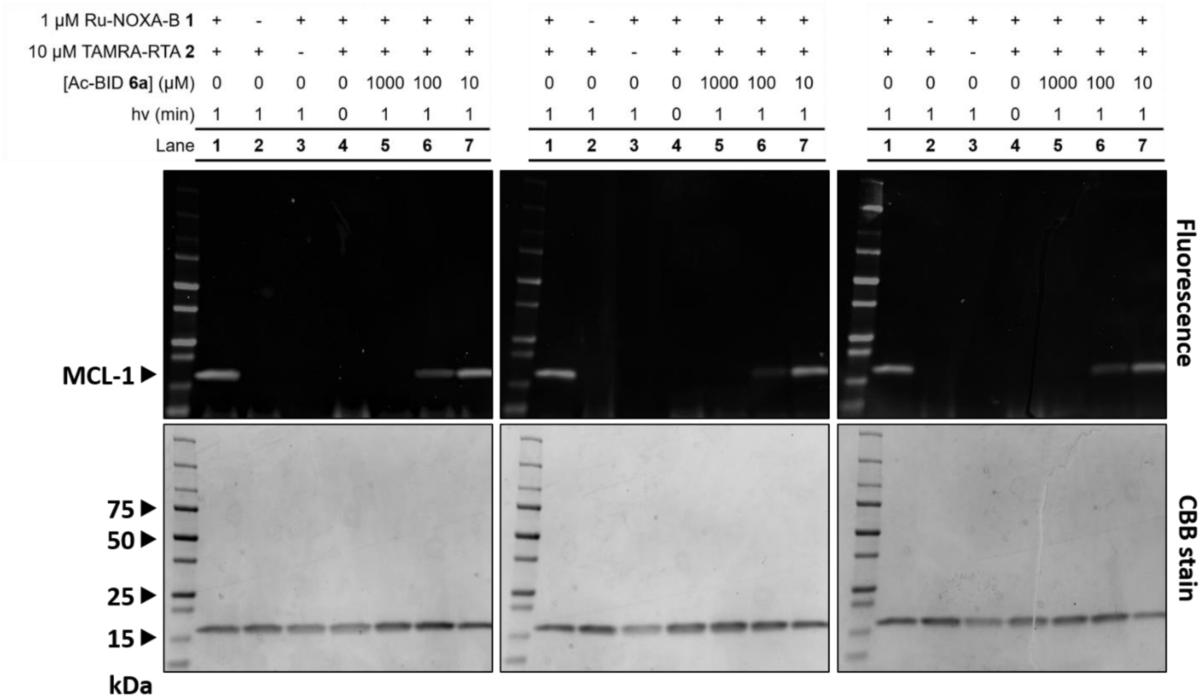

Supplementary Figure 28: *Full gels of competition experiments with Ac-BID* ***6a*** *displayed in Fig. 4 (3 independent repeats). Fluorescence image (top) and Coomassie Brilliant Blue (CBB, bottom) stained SDS-PAGE gels; fluorescence image shows that fluorescent labelling of MCL-1 mediated by Ru(II)(bpy)$_3$-NOXA-B* ***1*** *is suppressed by increasing concentrations of Ac-BID* ***6a*** *(lanes 5-7). Conditions: 5 μM MCL-1, 1 μM Ru(II)(bpy)$_3$-NOXA-B* ***1****, 10 μM TAMRA-RTA* ***2****, 10 μM APS, 1 min irradiation, 50 mM $(NH_4)HCO_3$ (pH 7.4).*

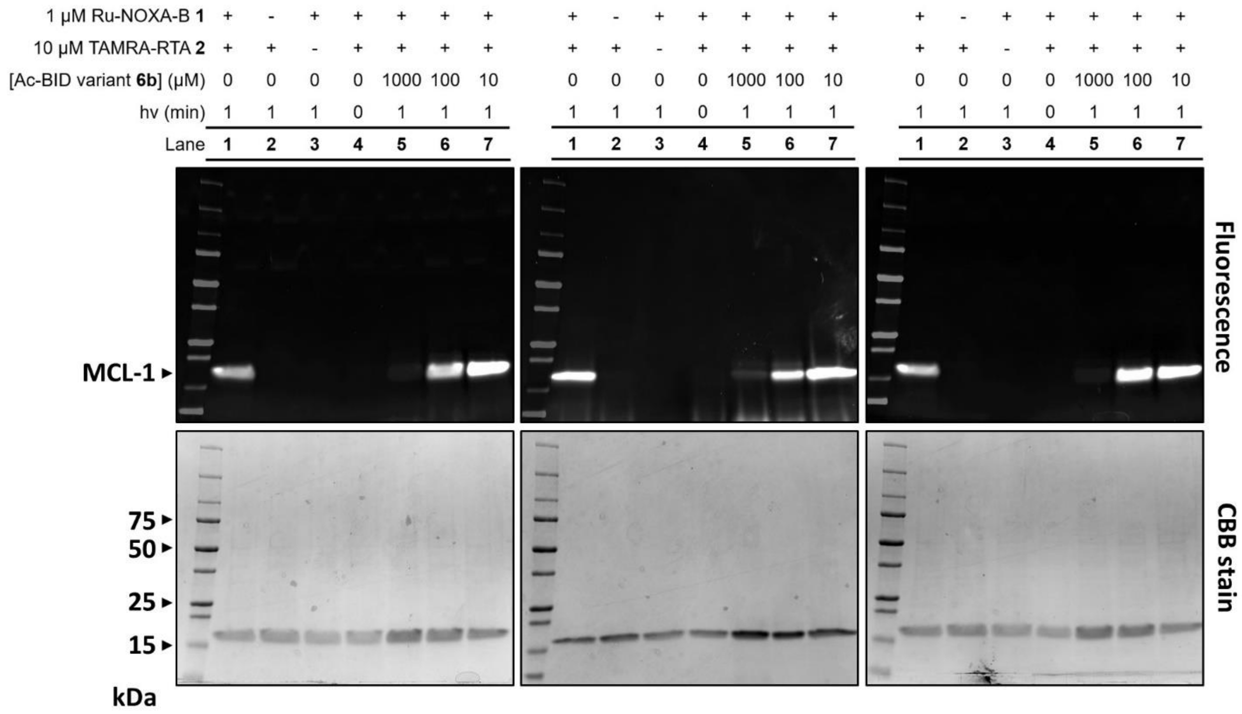

Supplementary Figure 29: *Full gels of competition experiments with Ac-BID variant **6b** displayed in Fig. 4 (3 independent repeats). Fluorescence image (top) and Coomassie Brilliant Blue (CBB, bottom) stained SDS-PAGE gels; fluorescence image shows that fluorescent labelling of MCL-1 mediated by Ru(II)(bpy)$_3$-NOXA-B **1** is suppressed by 1000 µM **6b** but not by 10 or 100 µM **6b** (lanes 5-7). Conditions: 5 µM MCL-1, 1 µM Ru(II)(bpy)$_3$-NOXA-B **1**, 10 µM TAMRA-RTA **2**, 10 µM APS, 1 min irradiation, 50 mM $(NH_4)HCO_3$ (pH 7.4).*

Competition experiments using Ac-BID in combination with <u>non-targeted SET reagent</u> Ru(II)(bpy)3

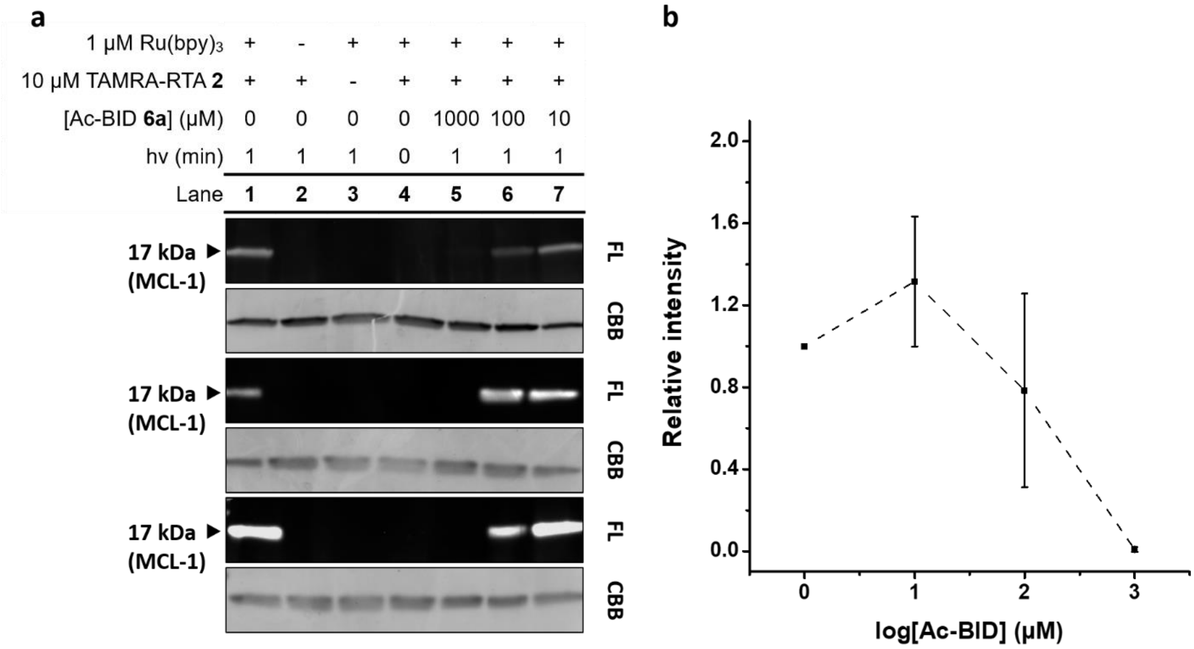

Supplementary Figure 30: *Competition experiments with Ac-BID **6a** and non-targeted SET catalyst Ru(II)(bpy)$_3$. (a) SDS-PAGE gel showing that fluorescent labelling of MCL-1 mediated by Ru(II)(bpy)$_3$ is suppressed by increasing concentrations of Ac-BID (lanes 5-7). Conditions: 5 μM MCL-1, 1 μM Ru(II)(bpy)$_3$, 10 μM TAMRA-RTA **2**, 10 μM APS, 0-1000 μM Ac-BID **6a**, 1 min irradiation, 50 mM $(NH_4)HCO_3$ (pH 7.4). Pictures of complete gels are shown in Supplementary Figure 27. (b) Plot of relative fluorescence intensity (intensity of fluorescent band (FL)/intensity of Coomassie Brilliant Blue (CBB) band; normalised to the experiment in lane 1) for the competition experiment with different concentrations of Ac-BID. Error bars represent the standard deviations from three independent repeats.*

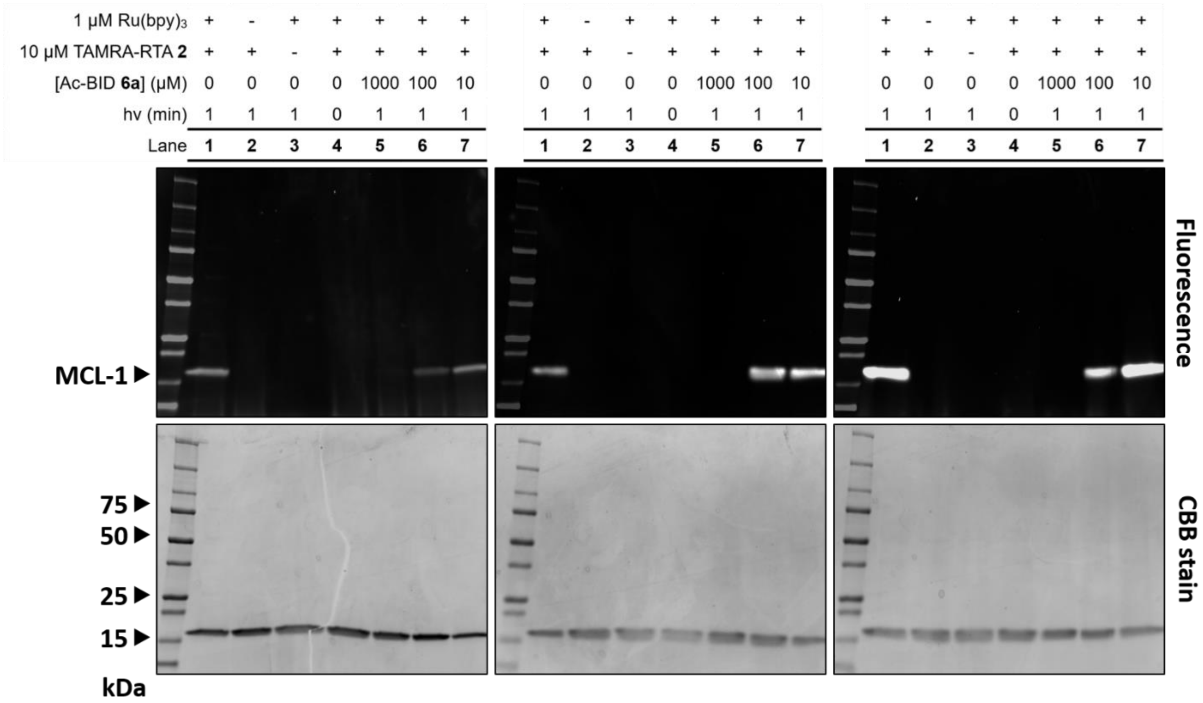

Supplementary Figure 31: *Full gels for competition experiments with Ac-BID* ***6a*** *and non-targeted SET catalyst Ru(II)(bpy)$_3$ displayed in Supplementary Fig. 26**.**. Fluorescence image (top) and Coomassie Brilliant Blue (CBB, bottom) stained SDS-PAGE gels; fluorescence image shows that fluorescent labelling of MCL-1 mediated by Ru(II)(bpy)$_3$ is suppressed by increasing concentrations of Ac-BID* ***6a*** *(lanes 5-7). Conditions: 5 µM MCL-1, 1 µM Ru(II)(bpy)$_3$, 10 µM TAMRA-RTA* ***2****, 10 µM APS, 1 min irradiation, 50 mM (NH$_4$)HCO$_3$ (pH 7.4).*

Selective labelling of MCL-1 with TAMRA-RTA over BCL-X_L

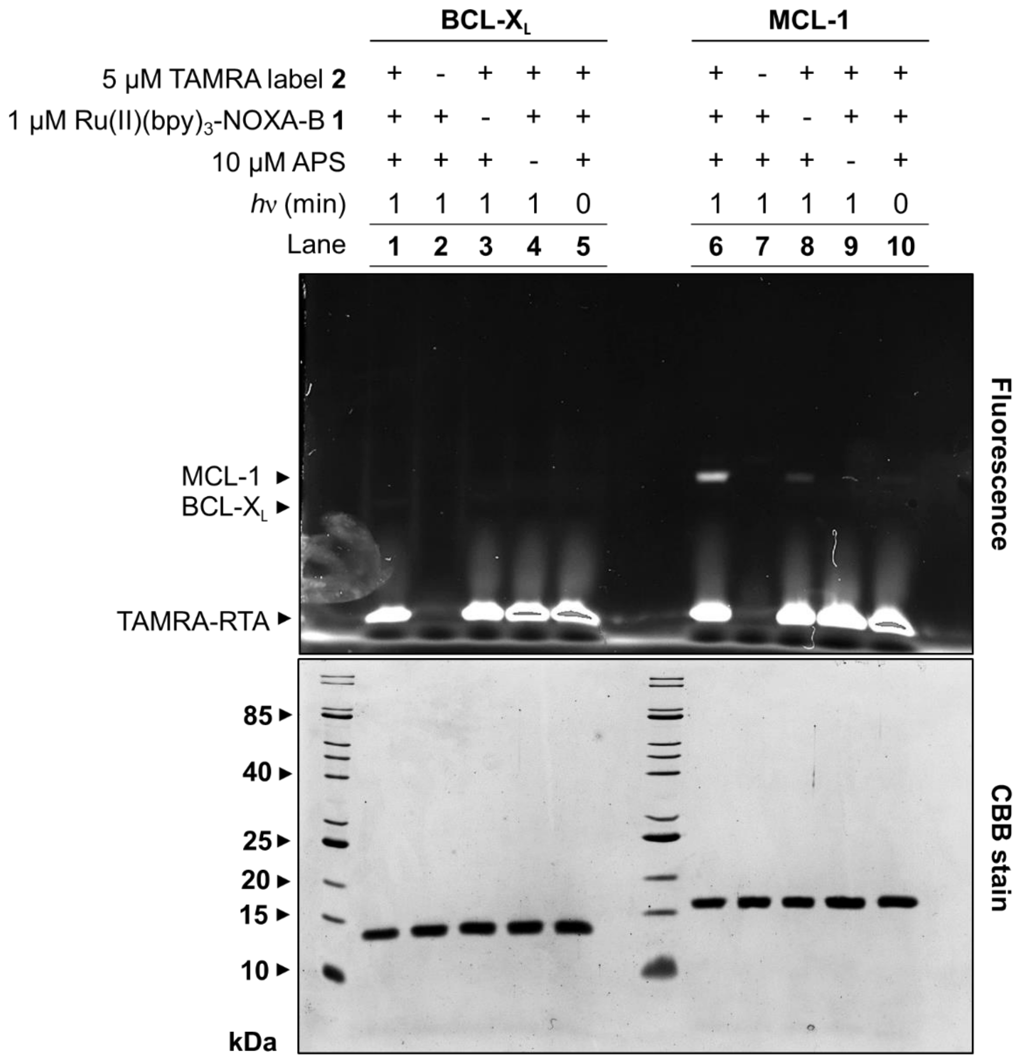

Supplementary Figure 32: *Photolabelling of BCL-X_L and MCL-1 with TAMRA-RTA **2**. Fluorescence image (top) and Coomassie Brilliant Blue (CBB, bottom) stained SDS-PAGE gels; fluorescence image shows only MCL-1 is labelled when 1 min hv, 5 µM TAMRA-RTA **2** and 1 µM Ru(II)(bpy)$_3$-NOXA-B **1** are present (5 µM MCL-1 and 10 µM APS used), CBB stain shows BCL-X_L and MCL-1 for all conditions.*

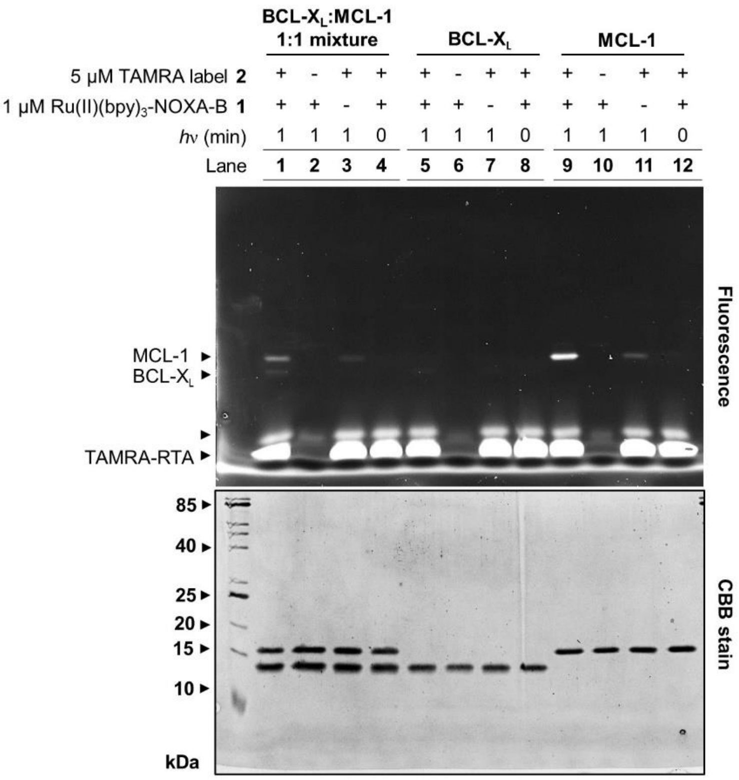
BCL-XL:MCL-1
1:1 mixture
BCL-XL
MCL-1
5 µM TAMRA label 2
1 µM Ru(II)(bpy)3-NOXA-B 1
hν (min)
Lane
1 2 3 4 5 6 7 8 9 10 11 12
MCL-1
BCL-XL
TAMRA-RTA
Fluorescence
85
40
25
20
15
10
kDa
CBB stain

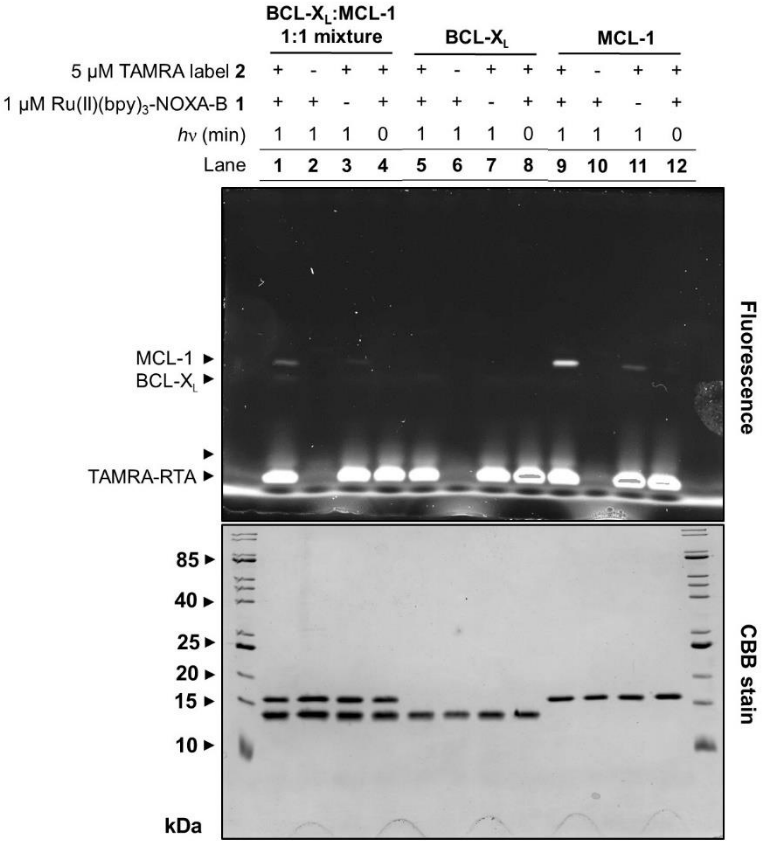
BCL-XL:MCL-1
1:1 mixture
BCL-XL
MCL-1
5 µM TAMRA label 2
1 µM Ru(II)(bpy)3-NOXA-B 1
hν (min)
Lane
1 2 3 4 5 6 7 8 9 10 11 12
MCL-1
BCL-XL
TAMRA-RTA
Fluorescence
85
40
25
20
15
10
kDa
CBB stain

Supplementary Figure 33: *Full gels for selectivity experiments with MCL-1 and BCL-X_L displayed in Fig. 5b (two independent repeats). MCl-1 is selectively labelled with TAMRA-RTA **2** over BCL-X_L in a 1:1 mixture of proteins, while BCL-X_L is not labelled in solution on its own. Conditions: 5 µM protein, 5 µM TAMRA-RTA **2** and 1 µM Ru(II)(bpy)$_3$-NOXA-B **1**, 10 µM APS, 1 min hv.*

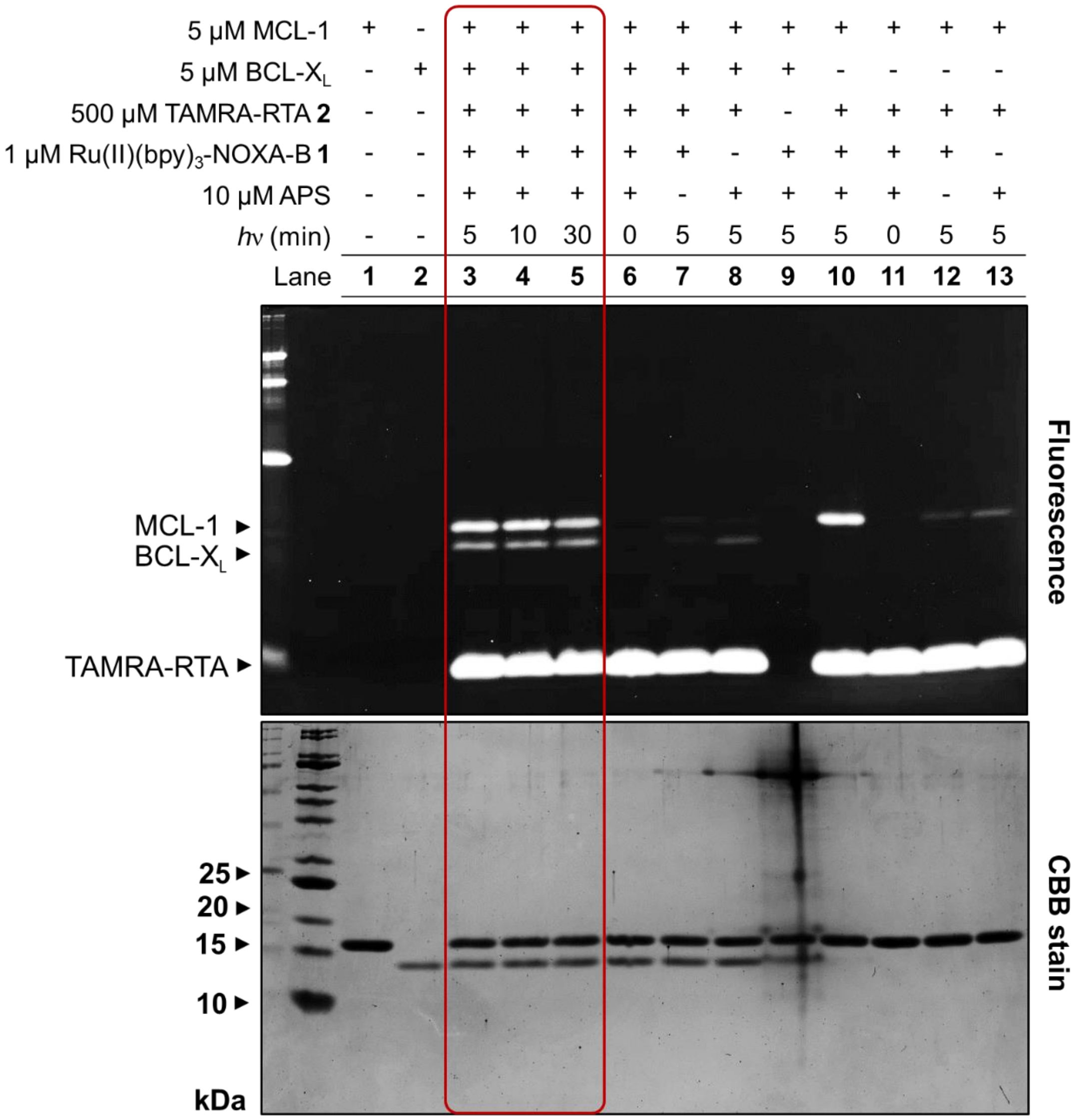

Supplementary Figure 34: *Both MCL-1 and BCL-X_L are labelled in a 1:1 mixture of proteins (lanes 3, 4 and 5) when high concentrations of TAMRA-RTA **2** are used. Conditions: 5 µM protein, 1 µM Ru(II)(bpy)$_3$-NOXA-B **1**, 500 µM TAMRA-RTA **2**, 10 µM APS, 50 mM $(NH_4)HCO_3$ (pH 7.4), 1 min hv.*

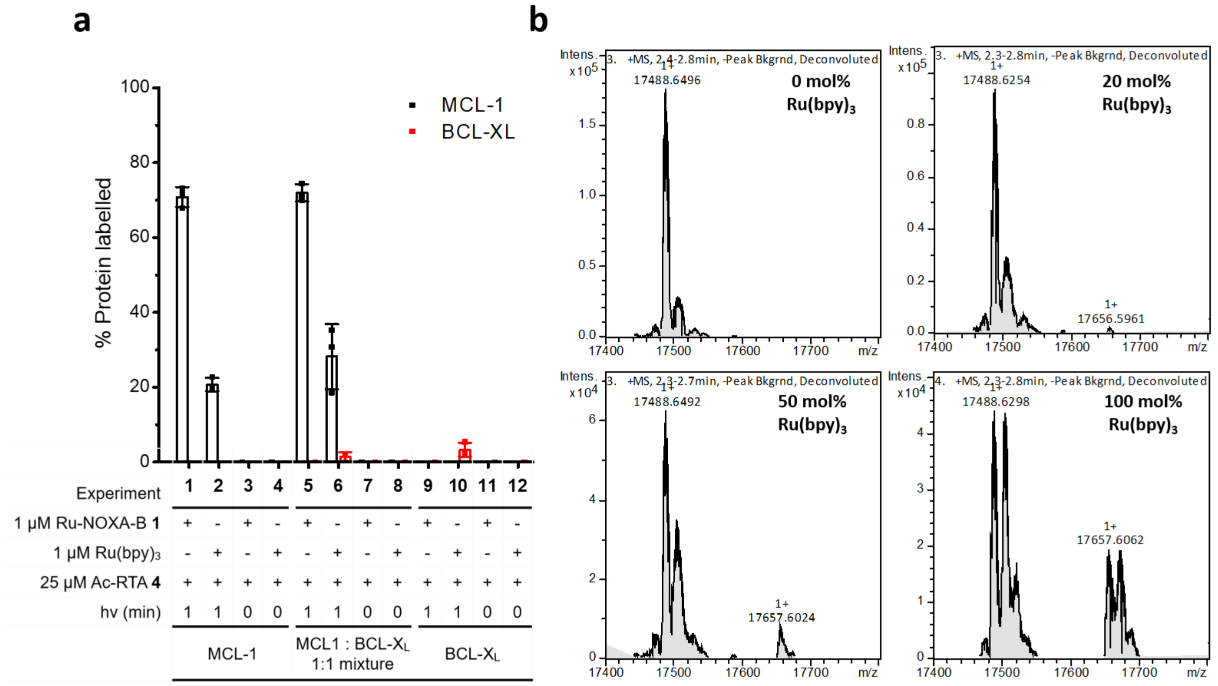

Supplementary Figure 35: *Photolabelling of MCL-1 and BCL-X_L with Ac-RTA **4**. (a) SET photolabelling mediated by Ru(II)(bpy)$_3$-NOXA-B **1** shows that MCL-1 (black bar) is selectively labelled with Ac-RTA **4** over BCL-X_L (red bar) in a 1:1 mixture of proteins, while BCL-X_L is not labelled in solution on its own. SET photolabelling mediated by Ru(II)(bpy)$_3$ shows that both MCL-1 and BCL-X_L are labelled with Ac-RTA **4**. Ru(II)(bpy)$_3$-mediated photolabelling of BCL-X_L was found to be less efficient than Ru(II)(bpy)$_3$-mediated photolabelling of MCL-1. Conditions: 5 µM protein, 1 µM photocatalyst (Ru(II)(bpy)$_3$-NOXA-B **1** or Ru(II)(bpy)$_3$), 25 µM Ac-RTA **4**, 10 µM APS, 1 min irradiation, 50 mM $(NH_4)HCO_3$ (pH 7.4). Error bars represent the standard deviations from three independent repeats. (b) Deconvoluted ESI-MS spectra showing increased SET photolabelling of BCL-X_L with Ac-RTA **4** (and increased BCL-X_L degradation) mediated by increasing concentrations of Ru(II)(bpy)$_3$. BCL-X_L (17488 Da) and photolabelled BCL-X_L (17657 Da). Conditions: 5 µM BCL-X_L, 0-5 µM Ru(II)(bpy)$_3$, 50 µM Ac-RTA **4**, 10 µM APS, 50 mM $(NH_4)HCO_3$ (pH 7.4).*

Selective labelling of MCL-1 with TAMRA-RTA over *h*DM2

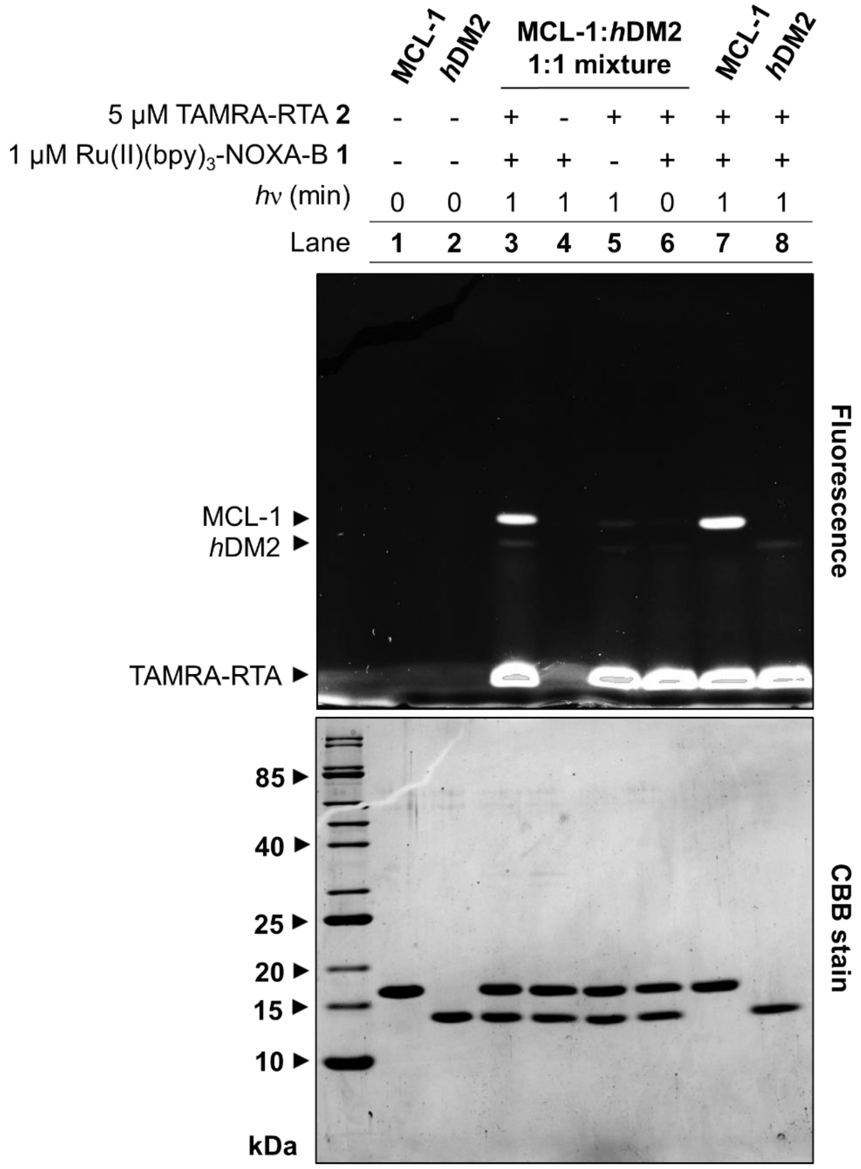

Supplementary Figure 36: *MCL-1 is selectively labelled in a 1:1 mixture of MCL-1 and hDM2 (lane 3). Conditions: 5 µM protein, 1 µM Ru(II)(bpy)$_3$-NOXA-B* ***1****, 5 µM TAMRA-RTA* ***2****, 10 µM APS, 50 mM $(NH_4)HCO_3$ (pH 7.4), 1 min hv.*

Supplementary References

1. Still, W. C., Kahn, M. & Mitra, A. Rapid chromatographic technique for preparative separations with moderate resolution. *J. Org. Chem.* **43**, 2923–2925 (1978).

2. Zhang, R. *et al.* Development of a ruthenium(II) complex-based luminescent probe for hypochlorous acid in living cells. *Inorg. Chem.* **52**, 10325−10331 (2013).

3. Sullivan, B. P., Salmon, D. J. & Meyer, T. J. Mixed phosphine 2,2'-bipyridine complexes of ruthenium. *Inorg. Chem.* **17**, 3334–3341 (1978).

4. Grison, C. M. *et al.* Double quick, double click reversible peptide "stapling". *Chem. Sci.* **8**, 5166–5171 (2017).

5. Miles, J. A. *et al.* Hydrocarbon constrained peptides – understanding preorganisation and binding affinity. *Chem. Sci.* **7**, 3694–3702 (2016).

6. Sato, S. & Nakamura, H. Ligand-directed selective protein modification based on local single-electron-transfer catalysis. *Angew. Chem. Int. Ed.* **52**, 8681–8684 (2013).

7. Fletcher, J. M. *et al.* De novo coiled-coil peptides as scaffolds for disrupting protein–protein interactions. *Chem. Sci.* **9**, 7656–7665 (2018).

8. Plante, J. P. *et al.* Oligobenzamide proteomimetic inhibitors of the p53–hDM2 protein–protein interaction. *Chem. Commun.* **34**, 5091–5093 (2009).

9. Barnard, A. *et al.* Orthogonal functionalisation of α-helix mimetics. *Org. Biomol. Chem.* **12**, 6794–6799 (2014).
